# Variability of cell wall recalcitrance and composition in genotypes of *Miscanthus* from different genetic groups and geographical origin

**DOI:** 10.3389/fpls.2023.1155188

**Published:** 2023-06-06

**Authors:** Rosario Iacono, Gancho T. Slavov, Christopher L. Davey, John Clifton-Brown, Gordon Allison, Maurice Bosch

**Affiliations:** ^1^ Institute of Biological Environmental and Rural Sciences (IBERS), Aberystwyth University, Gogerddan, Aberystwyth, United Kingdom; ^2^ Radiata Pine Breeding Company, Rotorua, New Zealand; ^3^ Department of Agronomy and Plant Breeding, Justus Liebig University Giessen, Giessen, Germany

**Keywords:** biomass, cell wall, genetic diversity, geographical distribution, germplasm, *Miscanthus*, pedoclimatic conditions, recalcitrance (saccharification)

## Abstract

*Miscanthus* is a promising crop for bioenergy and biorefining in Europe. The improvement of *Miscanthus* as a crop relies on the creation of new varieties through the hybridization of germplasm collected in the wild with genetic variation and suitable characteristics in terms of resilience, yield and quality of the biomass. Local adaptation has likely shaped genetic variation for these characteristics and is therefore important to quantify. A key biomass quality parameter for biorefining is the ease of conversion of cell wall polysaccharides to monomeric sugars. Thus far, the variability of cell wall related traits in *Miscanthus* has mostly been explored in accessions from limited genetic backgrounds. Here we analysed the soil and climatic conditions of the original collection sites of 592 *Miscanthus* genotypes, which form eight distinct genetic groups based on discriminant analysis of principal components of 25,014 single-nucleotide polymorphisms. Our results show that species of the genus *Miscanthus* grow naturally across a range of soil and climate conditions. Based on a detailed analysis of 49 representative genotypes, we report generally minor differences in cell wall characteristics between different genetic groups and high levels of genetic variation within groups, with less investigated species like *M. floridulus* showing lower recalcitrance compared to the other genetic groups. The results emphasize that both inter- and intra- specific variation in cell wall characteristics and biomass recalcitrance can be used effectively in *Miscanthus* breeding programmes, while also reinforcing the importance of considering biomass yield when quantifying overall conversion efficiency. Thus, in addition to reflecting the complexity of the interactions between compositional and structural cell wall features and cell wall recalcitrance to sugar release, our results point to traits that could potentially require attention in breeding programmes targeted at improving the *Miscanthus* biomass crop.

## 1 Introduction

Mitigating the effects of climate change while coping with shortages of fossil fuels is the challenge that defines our time and calls for shifting to renewable sources of fuel and chemicals. Plant biomass is an abundant source of building blocks for fuel and chemicals, thus having the potential to replace fossil feedstock for their production. Facilities dedicated to processing biomass to produce fuel, electricity, and chemicals are defined as biorefineries. However, to become a realistic alternative to petrol refineries, biorefineries require a constant supply of feedstock with optimal characteristics for the specific conversion processes (Hoang et al., 2015; Torres et al., 2019; Oyedeji et al., 2021; Brancourt-Hulmel et al., 2022).

Perennial biomass crops, ideally grown on lands not used for food production (e.g. marginal lands), represent attractive feedstocks for biorefining (Kang et al., 2013; Von Cossel et al., 2019; Yang et al., 2019; Pancaldi and Trindade, 2020). Perennial rhizomatous grass species of the genus *Miscanthus*, exhibiting C4 photosynthesis and widely distributed in East Asia with 16 species described in the wild (The Plant List, 2013), are one of the best candidates for the implementation of these cropping systems in Europe (Clifton-Brown et al., 2019a; Clifton-Brown et al., 2019b).

The cell wall, rich in sugar and aromatic molecules is the main energy and carbon sink in nature incorporating 45% of the fixed carbon (Barnes and Anderson, 2018; Tang et al., 2018). It is the main component of plant biomass and the one with the highest potential for producing chemicals and fuel (Albersheim et al., 2011). Its structure and composition depend on the organ considered (da Costa et al., 2017), the phenological stage (Rancour et al., 2012) as well as on the genetic background of the plant (da Costa et al., 2017). Importantly, being the dynamic interface between the plant and the external environment, the composition and structure of the cell wall can change in response to a plant’s exposure to external stressors (Moura et al., 2010; Domon et al., 2013; Le Gall et al., 2015; Vaahtera et al., 2019; Gladala-Kostarz et al., 2020). The interplay between the maintenance of cell wall integrity and reaction to environmental stresses has been reviewed recently by Baez et al. (2022).

A key biomass quality parameter for biorefining is the ease by which cellulosic and hemicellulosic cell wall polysaccharides can be converted to monomeric sugars (DeMartini et al., 2013). The complex and dynamic structure of the cell wall has evolved to maintain its functional integrity in response to developmental and environmental cues. This has resulted in the natural recalcitrance of the cell wall to deconstruction (McCann and Carpita, 2015), which hinders the profitable use of biomass for the production of fuel and chemicals (DeMartini et al., 2013).

*Miscanthus* species can intercross and produce sterile hybrids (Tamura et al., 2016) and the use of hybridization between selected lines has been proposed as a suitable breeding technique to develop *Miscanthus* into a specialized bioenergy and biorefining crop (Lewandowski et al., 2016). In the last decade, this approach led to the development of new hybrids (Kalinina et al., 2017; Clifton-Brown et al., 2019a; Clifton-Brown et al., 2019b) commercialized by companies such as Terravesta (Lincoln, UK). The hybridization technique to create new hybrids relies on the existence of a sufficient range of intra- and inter-specific variability in the target traits and on the ability to identify the most promising parental lines expressing them. However, the extent of variability for cell wall traits in the genus *Miscanthus* has mostly been explored in the natural hybrid *M.* × *giganteus* and its parent species *M. sacchariflorus* and *M. sinensis* (Lygin et al., 2011; van der Weijde et al., 2017b; da Costa et al., 2019; Bilska-Kos et al., 2022). There is little information on the variation in cell wall related traits between *Miscanthus* genotypes from a wider range of different genetic groups.

The process of selecting parental lines for the production of hybrids starts with the collection of promising germplasm in the wild. This phase is time-consuming and expensive. Notably, the structure and composition of the cell wall are presumably adapted to the environmental conditions where the genotype was originally collected. Thus, geographical information plays an important role in the process of selection of germplasm for the creation of varieties with a superior biomass quality for conversion. For instance, Li et al. (2016) found that there is a relation between the geographical area where a *Miscanthus* accession was sampled and the amount of glucose released with enzymatic digestion after acid or alkali pretreatment.

In this context, spatial analysis using geographic information system (GIS) technology (Hyman et al., 2013) can assist plant breeding by uncovering the environmental associations of germplasm across a wide range of collection sites (Hijmans and Spooner, 2001). Breeders routinely use spatial analysis (i.e., either explicitly or implicitly) to inform the decision about where to test and disseminate crop varieties (Oshunsanya and Aliku, 2016) and to identify interesting starting materials such as for the resilience to abiotic stress in wild accessions (Hijmans and Spooner, 2001). For example, Malinowska et al. (2017) found that a model based on the precipitation and temperature conditions in the area of origin of *Miscanthus* genotypes can predict their resistance to drought.

Here, we focused on *Miscanthus* genotypes grown in a spaced field trial and used analysis of single-nucleotide polymorphism (SNP) data and the pedo-climatic conditions of their original collection sites to select representatives belonging to broadly diverse and distinct genetic groups. We demonstrate the value of using environmental data to identify sites where germplasm of the genus *Miscanthus* with suitable characteristics can be found, with the potential to assist breeding programmes. We report differences in cell wall characteristics between and within the different genetic groups of *Miscanthus*, including for recalcitrance to enzymatic sugar release, identify compositional and structural features that correlate with cell wall recalcitrance and discuss the practical implications of our findings.

## 2 Materials and methods

### 2.1 Plant material, genetic groups and sample preparation

#### 2.1.1 Experimental field and origin of *Miscanthus* genotypes

Genotypes of *Miscanthus* were selected from the ABR33 replicated field trial established between 2012 and 2014 near Aberystwyth (Wales, UK; 52° 25’ 57.7” N - 4° 01’ 33.2” W). Briefly, 953 *Miscanthus* accessions (i.e., genotypes), which had previously been brought into and grown across Europe, were propagated vegetatively using rhizome division and planted in a Randomised Complete Block Design, with one replicate per genotype in each of three blocks planted at 1.5 × 1.5 m spacing. In addition to previously established trials near Aberystwyth, rhizome propagules had been collected from trials near Catania (Sicily, Italy) and Braunschweig (Lower Saxony, Germany). The original source geographic coordinates were available for 592 out of the 953 genotypes in ABR33 and were downloaded from the IBERS MScan Database (Huang et al., 2019). These 592 Miscanthus genotypes were originally collected from an area between 18° 30’ 0’’ N - 109° 18’ 18’’ E and 45° 12’ 25.92’’ N - 144° 26’ 42’’ E and included South-East China, South Korea, Taiwan, and Japan. In terms of altitude, the genotypes were collected from sea level to 3000 m above sea level.

#### 2.1.2 Identification of genetic groups

**Figure 1:**
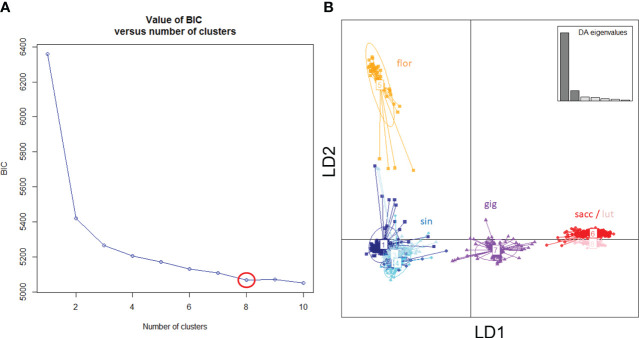
Genetic groups of *Miscanthus* in the ABR33 field trial based on Discriminant Analysis of Principal Components (DAPC). **(A)** Inference of ‘optimal’ number of groups based on the rate of change of model Bayesian Information Criterion (BIC). **(B)** Scatter plot of the first two linear discriminants (LD1 and LD2), with groups labelled using *a priori* species designation: sin = *Miscanthus sinensis*; flor = *M. floridulus*; sacc = *M. sacchariflorus*; lut = *M. lutarioriparius*; gig = *M. × giganteus* and inset showing the eigenvalues of discriminant analysis (DA).

Leaf samples were collected from all 953 genotypes that survived through the spring of 2015 and DNA was extracted using previously described protocols (Slavov et al., 2013a). RAD-Seq genotyping was then performed by Floragenex as described by Slavov et al. (2014). Because several species of *Miscanthus* were sampled (**Figure 1**), and reference genome data was only available for some of these, RAD-Seq reads were aligned and single nucleotide polymorphism (SNP) genotyping data generated using the reference-free UNEAK pipeline (Lu et al., 2013), which is particularly suitable for species with highly repetitive and complex genomes, such as *Miscanthus* (Mitros et al., 2020; De Vega et al., 2021; Zhang et al., 2021). The default parameters of UNEAK were used and data was exported for 25,014 SNPs that had minor allele frequencies of at least 0.0025 (i.e., at least 5 minor allele copies) and call rates of at least 80%. To define genetic groups objectively, the resulting SNP data were subjected to discriminant analysis of principal components (DAPC) using the *adegenet* R package (Jombart, 2008; Jombart et al., 2010), and the ‘optimal’ number of groups was selected using the *find.clusters* function within the *adegenet* R package.

#### 2.1.3 Sample collection and processing

Sample collection and processing was performed as described by Huang et al. (2019) with some minor modifications. Briefly, above-ground biomass was collected in early spring 2016 from 49 completely senesced *Miscanthus* genotypes across different genetic groups, with three biological replicates for each genotype. At the time of sampling, the plants were 4 years old. The cut biomass (comprised of stem and leaf material) was weighed to determine the total fresh weight. A sub-sample of approx. 200 g was removed and its relative moisture content was determined after drying at 60°C to constant weight. The percentage of moisture was used to calculate the approximate dry weight per harvest per plant. Dried samples were ground to a 1.5 mm mesh and stored at room temperature until further processing. Yield data for the biomass harvested for each genotype in 2016 was obtained from the MScan database at IBERS.

#### 2.1.4 Cell wall preparation

All compositional analyses were carried out on purified cell walls. Approximately 70 mg of the 1.5 mm mesh biomass samples were weighed in 2 mL microtubes (Sarstedt, Cat. 72.609.001) along with two stainless steel balls and positioned in a Plant Grinding and Preparation System from Labman^®^ similar to the one described by Santoro et al. (2010).

Cell wall material was extracted as described by da Costa et al. (2014) with minor modifications. The efficacy of the extraction at several steps was tested, as suggested by Fry (1988). Cell wall purification was performed using an alcohol-insoluble residue (AIR) preparation followed by starch removal. Preparation of AIR started with the addition of 1.5 mL of 70% v/v ethanol, followed by thorough vortexing (2400 rpm x 30 sec) and incubation for 16 h in a shaking incubator set at 40°C and 150 rpm. Samples were then centrifuged at 10,000 rpm to pellet the residue. The pellet was repeatedly washed with 70% v/v ethanol until a stable A280 reading of the supernatant. Subsequently, the pellet was washed twice by adding 1.5 mL of a chloroform/methanol (1:1) solution, vortexed to resuspend the pellet, and incubated for 30 min at 25°C and 150 rpm. After each wash, samples were centrifuged at 10,000 rpm for 10 min and the supernatant was discarded. Finally, the pellet was washed three times with 1.5 mL of 100% acetone with resuspension by vortexing, incubation, centrifugation, and discarding of supernatant performed as in the previous step. The samples were then dried under airflow at room temperature. After this step, dry samples were stored at room temperature until further processing.

Starch was removed using α-amylase from the porcine pancreas (Novozyme E-PANAA-3G), following the method described by Foster et al. (2010) as adapted by da Costa et al. (2014), with minor additional modifications. Briefly, starch removal was initiated by re-suspending the AIR pellet in 1.5 mL of 0.1 M sodium acetate buffer pH 5. Tubes were capped and incubated at 80°C for 20 min to gelatinize the starch. Afterwards, samples were cooled on ice, centrifuged at 10,000 rpm, and the supernatant was discarded. The resulting pellet was washed three times with 1.5 mL of H_2_O. Next, 1.5 mL of a mixture containing 0.3 µL of 1% w/v sodium azide, 15.6 µL of porcine α-amylase (3000 U mL^-1^) and 1.4841 mL of H_2_O were added to each sample. Samples were incubated in a shaking incubator for 48 h at 35°C and 110 rpm. The digestion was terminated by heating the samples to 95°C for 15 min, and the samples were subsequently cooled on ice. Finally, samples were centrifuged (10,000 rpm for 10 min), and the supernatant was discarded. Pellets were washed three times with water and two times with 100% acetone, as described previously, and dried at room temperature under a gentle flow of air. The absence of starch was confirmed using Lugol’s staining test as described by Barnes and Anderson (2017).

### 2.2 Acid hydrolysis for total monosaccharide release

The total monosaccharide composition of cell wall material was determined by double hydrolysis (Saeman, 1945; Sluiter et al., 2008; Pettolino et al., 2012; Petit et al., 2019). Approximately 10 mg of the previously prepared cell wall material was weighed into 10 mL Pyrex glass tubes fitted with polypropylene caps. Subsequently, 0.100 mL of 72% (w/w) H_2_SO_4_ was added, and the tubes were capped and placed on a heating block set at 30°C for 60 min, during which time the samples were vortexed every 5 to 10 minutes. A set of sugar recovery standards (SRS) was prepared and taken through the remaining hydrolysis to correct for losses due to the destruction of sugars during dilute acid hydrolysis. Subsequently, deionized water was added to obtain a 4% (w/w) H_2_SO_4_ solution, and samples were mixed to eliminate phase separation. The sealed tubes were placed in an autoclave at 121°C for 1 h. Once at room temperature, the tubes were centrifuged to produce a particulate-free supernatant, and the samples were diluted ten-fold by taking 0.100 mL of each sample and mixing it with 0.900 mL of deionized water. Samples were stored at −20°C until analysis. Just before analysis, samples were diluted 1 to 200 in ultrapure water and enzymatically released amounts of glucose (Glc), xylose (Xyl), and arabinose (Ara) were quantified using the high-performance anion exchange chromatography system as described below in section 2.4.

### 2.3 Saccharification analysis

Enzymatic release of monosaccharides was performed as described by Resch et al. (2015) and modified by da Costa et al. (2015) and Petit et al. (2019). Briefly, approximately 10 mg of cell wall material was manually weighed out in 2 mL polypropylene microtubes with a screw cap (Sarstedt, Cat. 72.609.001). The exact sample weight was recorded. Then, 0.3 mL of 100% acetone was added to each sample to collect the material at the bottom of the tube. Acetone was left to evaporate under a stream of air overnight. Next, 1 mL of saccharification mixture was added having the following composition: 0.957 mL of 0.025 M potassium acetate buffer (pH5.6), 0.0024 mL of cellulase from *Trichoderma reesei* (Cellulase, Sigma Aldrich, code C2730), 0.0006 mL of *β*-glucosidase from *Aspergillus niger* (Novozyme 188, Novozyme, discontinued), and 0.040 mL of 1% sodium azide to repress bacterial growth. The mixture was prepared in a single batch and kept at 4°C until use. Samples were incubated for 48 h at 50°C in a shaking incubator set at 150 rpm. Subsequently, samples were centrifuged at 10,000 rpm for 5 min, and 0.9 mL of the supernatant was transferred to a new 2 mL polypropylene tube and stored for 2-3 days at −20°C until monosaccharide quantification. Just before analysis, samples were diluted 1:50 in ultrapure water and enzymatically released amounts of glucose, xylose, and arabinose were quantified using the high-performance anion exchange chromatography system as described below in section 2.4.

### 2.4 Monosaccharide quantification

Monosaccharides in the solutions were separated and quantified by high-performance anion exchange chromatography (Thermo Fisher Scientific Inc. ICS-5000) coupled with pulsed amperometric detection (HPAEC-PAD) operated at 45°C using a CarboPac SA10 (4×250 mm) column with a CarboPac SA10G (4×50 mm) guard column. An eluent generator coupled to the system continuously prepared a KOH solution at 0.001 M for isocratic elution at a flow rate of 1.5 mL/min for 14 min. A volume of 0.025 mL of the sample was injected into the column and detected by PAD using a gold working electrode and an Ag/AgCl reference electrode. A set of calibration standards was prepared. The calibration curve was validated between 5 μg/mL and 40 μg/mL. Immediately before the HPAEC-PAD analysis, 0.080 mL of each 1:10 diluted sample was neutralized by adding 0.320 mL of 0.02 M KOH. Samples were further diluted 1:4 with deionised water, to a final dilution of 1:200 (da Costa et al., 2015). Aliquots of 0.400 mL of the diluted samples were then filtered through 0.45 μm nylon filter vials (Thomson SINGLE STEP; Thomson Instrument Company, Oceanside, California, USA). The Chromeleon software (v. 7.1; Thermo Fisher) was used for data processing. External calibration standards were used to identify and quantify the three most prominent monosaccharides detected in the chromatograms: glucose (Glc), xylose (Xyl), and arabinose (Ara).

### 2.5 Near infrared spectroscopy data

For each genotype, the content of cellulose, hemicellulose and lignin predicted by near-infrared spectroscopy (NIR) was determined by Analytical Chemistry (IBERS, Aberystwyth University) following procedures described by Allison et al. (2011).

### 2.6 Geo-environmental data sourcing

#### 2.6.1 Genotype geographical origin details

Based on data from MScan, a complete list of the accessions included in the ABR33 trial was compiled. For accessions that were originally collected in the wild, source geographic coordinates, as determined using the Global Positioning System (GPS), were downloaded in a comma-separated file format.

#### 2.6.2 Species distribution in the wild

The distribution of *Miscanthus* species in the wild was mapped using data from the Global Biodiversity Information Facility (GBIF) (Gbif.Org, 2019). The GBIF database contains data on species observations, including exact coordinates, from different sources. Data were standardized between various sources using the Darwin Core Standard (Wieczorek et al., 2012). The number of species observations per geographic point was counted and mapped using the R programming language (R Core Team, 2018).

#### 2.6.3 Climatic data

Temperature-related and precipitation-related bio-climatic variables (**Table S1**) were retrieved from the WorldClim database v.1.4 (Hijmans et al., 2005) for all georeferenced genotypes using R as described by Hijmans and Elith (2013). The resulting dataset was downloaded in comma-separated format.

#### 2.6.4 Soil data

Chemical and physical properties of the topsoil (TS, 0 – 100 cm from ground level) and the subsoil (SS, >100 cm from ground level) for each sampled location were downloaded from the Harmonized World Soil Database (HWSD) (Fao/Iiasa/Isric/Isscas/Jrc, 2009) in Microsoft Access format, then converted to a SQL 3 format using the MS Access to Sqlite3 Converter software (https://github.com/sanandrea/mdb2sq3). The resulting data was downloaded into a comma-separated file. **Table S2** shows an overview of the soil variables.

#### 2.6.5 Geo-environmental data analysis

Principal Component Analysis (PCA) was used to identify the traits with the largest contribution to the overall variability and consequently select genotypes (Maji and Shaibu, 2012) using the *get_pca_var* function in the factoextra package of R (R Core Team, 2018).

### 2.7 Statistical analysis of cell wall characteristics

Linear mixed-effect models (LMEMs) were used to test the effect of genetic group on the cell wall characteristics quantified in this study. We chose to use LMEMs as opposed to conventional analyses of variance (ANOVA) because of the unbalanced design of our experiment (i.e., different numbers of genotypes in different genetic groups), heteroscedasticity revealed by exploratory plots, and potential lack of independence of genotypes within genetic groups (i.e., because of coancestry). In LMEMs aimed at identifying contrasts among groups, genetic group was treated as a fixed effect and genotype and biological replicate as random effects. We also used LMEMs with all effects treated as random to partition the overall trait variance into group, genotype, and environmental sources (i.e., biological replicate and residual). All LMEMs were fitted using the *lmer* function in the lmerTest R package (Kuznetsova et al., 2017), which is an extended version of the same function in the widely used lme4 package (Bates et al., 2015). To identify significant contrasts, LMEMs with genetic group treated as a fixed effect were then passed on to the *emmeans* function from the emmeans R package (Lenth, 2022). Finally, results from these contrasts were visualized using a compact letter display obtained using the *cld* function from the R package multcomp (Hothorn et al., 2008). The datasets and the code used for the statistical analysis presented in this paper are available at: https://github.com/RosarioIacono/Iaconoetal2023Data.

## 3 Results

### 3.1 Genetic groups and geospatial distribution of *Miscanthus* genotypes

Based on results from DAPC, the ‘optimal’ number of groups was set at eight (i.e., there was no further reduction of the Bayesian Information Criterion with higher numbers of groups, **Figure 1**). *M. sinensis* was the only species represented by multiple groups (*n* = 4, **Table 1**). As expected, *M.* × *giganteus* formed a separate group with intermediate clustering between its parental species (*M. sinensis* and *M. sacchariflorus*).

**Table 1:** List of genetic groups and their ID as used in this paper.

Group ID	Genetic group
M1	*M. sinensis* South Japan
M2	*M. sinensis* (EMI/PRI)
M3	*M. sinensis* North Japan
M4	*M. sinensis* Taiwan
M5	*M. floridulus*
M6	*M. sacchariflorus/robustus*
M7	*M. × giganteus*
M8	*M. lutarioriparius*

**Figure 2:**
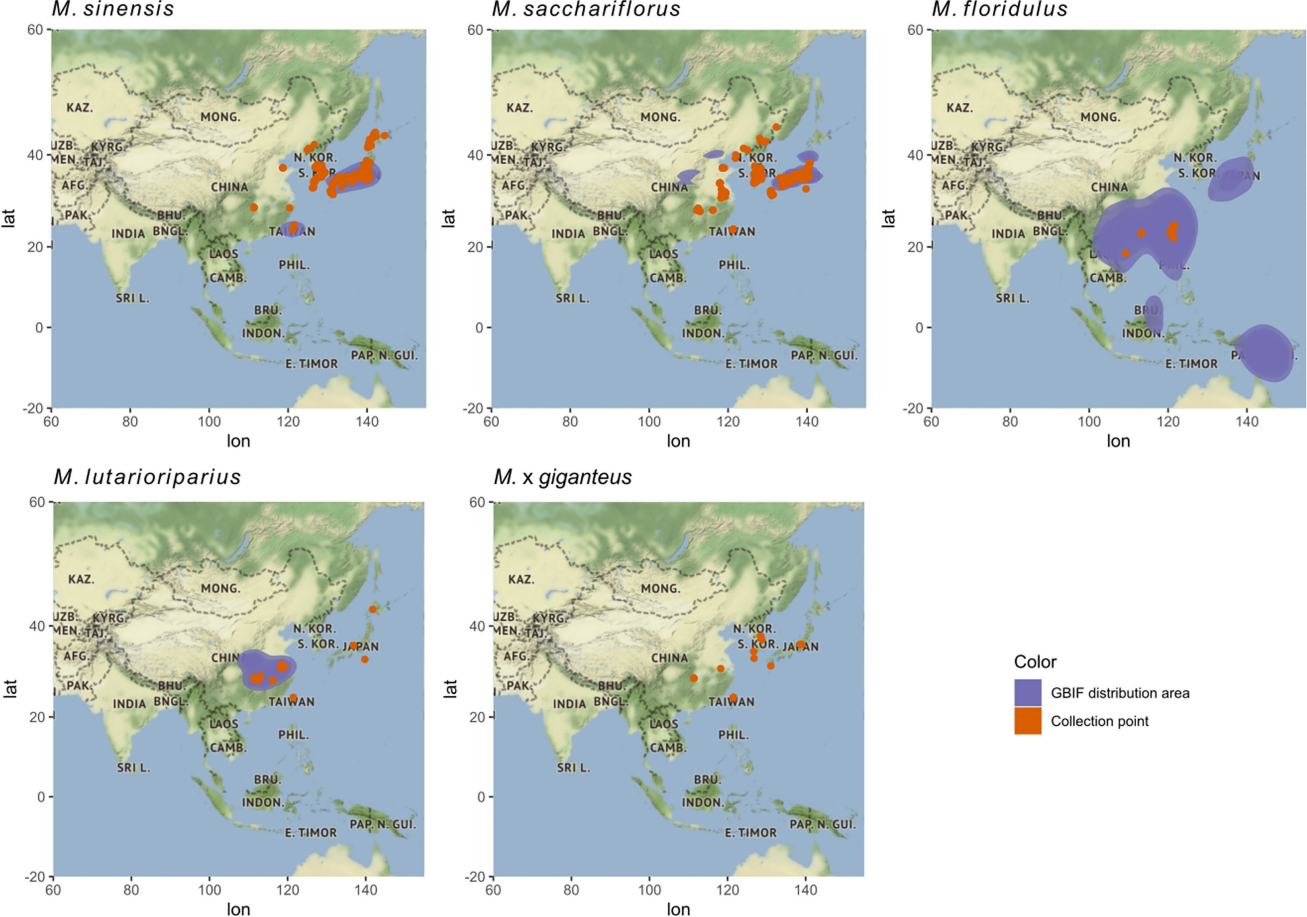
Area of distribution of *Miscanthus* species in the wild and collection points for genotypes in ABR33. The distribution of *Miscanthus* species is presented using data from the Global Biodiversity Information Facility (GBIF). The x-axis and y-axis indicate longitude and latitude, respectively. Blue shading represents the areas where each species has been reported in the wild according to the GBIF database, while the red dots represent the collection sites of genotypes in the ABR33 trial.

The four species of *Miscanthus* included in this study (*M. sacchariflorus*, *M. sinensis*, *M. floridulus*, and *M. lutarioriparius)* are distributed across a broad geographic area (blue shading in **Figure 2**). The distribution of the 592 *Miscanthus* genotypes included in the ABR33 trial (red dots in **Figure 2**) fell within the distribution of their corresponding genetic group reported in the GBIF database. Despite the extensive distribution overlap between *M. sacchariflorus* and *M. sinensis*, collection points of both *M. sacchariflorus* and *M. sinensis* in mainland China were recorded in areas where there was no GBIF report of the two species in the wild (red dots outside of the blue area), corroborated by collection points of inter-specific *M.* × *giganteus* hybrids in close proximity (**Figure 2**). In addition, *M. floridulus* and *M. lutarioriparius* genotypes were collected from a relatively small part of their respective distribution areas (**Figure 2**). Taken together, these observations point to the opportunity offered by databases like the one from GBIF to inform future germplasm collection campaigns and to the presence of vast unexplored areas where interesting new accessions could be found. Moreover, they highlight the possibility that more inter-specific hybrids, similar to commercially grown *M.* × *giganteus*, could perhaps be found with targeted collection campaigns in areas of coexistence of different species.

### 3.2 Pedo-climatic conditions

Using the coordinates for each collection point of the 592 *Miscanthus* genotypes, it was possible to retrieve a complete series of climatic and soil variables from the WorldClim database (Fick and Hijmans, 2017) and the Harmonised World Soil Database (Fao/Iiasa/Isric/Isscas/Jrc, 2009), respectively. The data describing climatic conditions over the last 50 years and soil conditions of the sites of origin of the 592 genotypes resulted in a multivariate dataset comprising 51 variables. The list and description of the variables are available in **Tables S1**, **S2**. The high number of variables and the well-known interdependence between precipitation, temperature, and pedologic conditions in specific environments suggested that the dataset may not be of full rank and some variables were partly redundant. To visualize the relations between the environmental variables we calculated the correlation between the variables in the soil-climate dataset (**Figure S1**). These correlations indicated that *Miscanthus* species can grow across a wide range of conditions in terms of salinity, seasonality of precipitation and soil composition.

### 3.3 Selection of 49 genotypes

**Figure 3:**
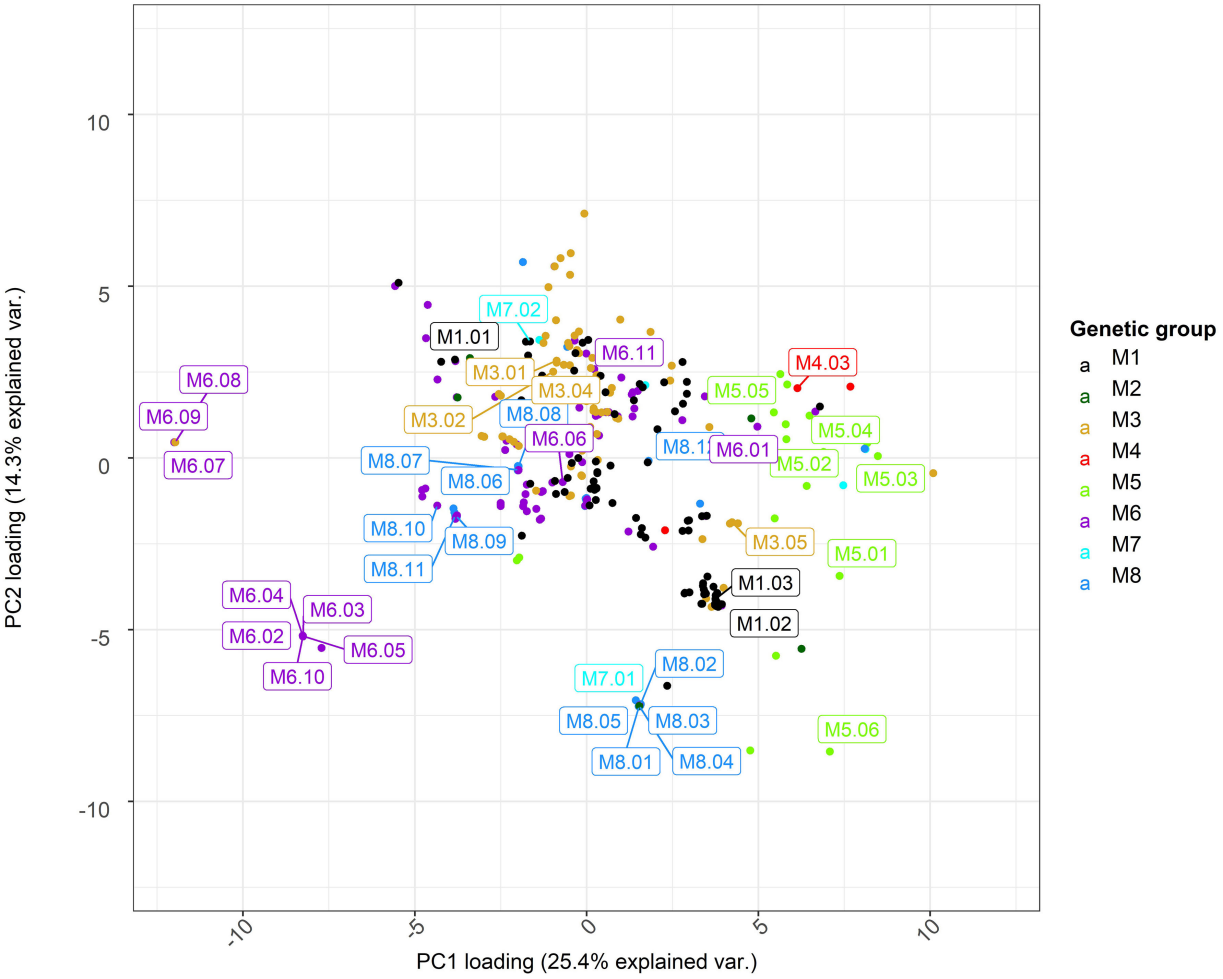
Principal component analysis (PCA) of climate and soil variables for sampling locations of ABR33 genotypes. Scores for the first and second principal components (PC1 and PC2) are shown along the x- and y-axis, respectively, with proportions of variance explained shown in parentheses. Labels indicate genotypes selected for biomass characterization. The loadings for PC1 and PC2 are shown in **Figure S2**. M1 = *M. sinensis* from South Japan, M2 = *M. sinensis* EMI/PRI, M3 = *M. sinensis* from North Japan, M4 = *M. sinensis* from Taiwan, M5 = *M. floridulus*, M6 = *M. sacchariflorus/robustus*, M7 = *M. × giganteus*, M8 = *M. lutarioriparius*.

We selected a number of representative genotypes to examine the cell wall properties of *Miscanthus* across different source locations and genetic groups. The pedo-climatic PCA score plot is presented in **Figure 3** and the plot of PCA loadings is shown in **Figure S2**. The first two components of the PCA model explained 40% of the total variance in the dataset. The first principal component (explaining 25.4% of the model variability) separated the *M. floridulus* group (mostly positive scores of green dots) from the other groups, particularly *M. sacchariflorus* (purple dots; **Figure 3**).

Analysis of the loadings shows that the inverse relation between the amount of precipitation (PTA, positive) and soil pH (T_PH and S_PH, negative) at *Miscanthus* collection sites are major contributors to the ordination along PC1 (**Figure S2**). Accessions in the *M. floridulus* group tended to be found in environments with high precipitation and low pH, while accessions of the *M. sacchariflorus* group were collected from environments characterized by a low amount of precipitation and higher values of pH in the soil. It is worth noting that pH is one of the main factors affecting soil nutrient availability. Soils with low pH generated under high precipitation conditions tend to have lower nutrient availability.

The selection of 49 representative *Miscanthus* genotypes was performed taking into account the PCA and genetic grouping results described above. We selected 35 genotypes from 7 genetic groups (i.e., maximizing environmental variation captured within each group) occurring in the wild and completed the selection with 14 genotypes which included naturally occurring and artificially generated (i.e., at IBERS) *M. × giganteus* hybrids (Hodkinson and Renvoize, 2001). Details of the 49 selected genotypes are shown in **Table S3**.

### 3.4 Differences in cell wall composition and characteristics among genetic groups

The main cell wall monosaccharides were quantified following acid hydrolysis of purified cell wall material of the 49 *Miscanthus* genotypes. The average content of glucose (Glc), xylose (Xyl) and arabinose (Ara) across the 49 genotypes was 39%, 28% and 2.5% (cell wall material (CWM); **Figure S3**), respectively, and in accordance with values previously reported for *Miscanthus* (e.g.: da Costa et al., 2017; van der Weijde et al., 2017b). Based on LMEM analyses, there were significant genetic group effects on the total content of Glc, Xyl and Ara in the cell wall (**Tables 2**, **S5**). However, group differences only accounted for 9-22% of the total variance and were mostly driven by contrasts with *M. floridulus* (**Figure S3**). Furthermore, variation among genotypes within groups was generally low and was only significant for Glc (14% of total variance), with most of the variation (56-79%) explained by the residual LMEM terms (**Table 2**).

**Table 2:** Variance components associated with genetic group (σ^2^_Group_), genotypes within groups (σ^2^_Geno_), biological replicates (σ^2^_Rep_), and residual error (σ^2^_Err_).

	Random Effects percentage			
	σ^2^_Group_	σ^2^_Geno_	σ^2^_Rep_	σ^2^_Err_
Glc	9.6	13.6	21.3	55.6
Xyl	21.5	6.7^n.s.^	10.3	61.4
Ara	8.7	3.3^n.s.^	9.1	78.9
NDF	45.8	35.0	0.3^n.s.^	18.9
ADF	49.0	32.0	2.4	16.6
ADL	36.4	38.7	1.9	23.0
Ash	48.0	25.0	1.6	25.4
DM	19.9	4.2^n.s.^	3.7^n.s.^	72.2
K Lignin	18.7	53.2	1.8	26.3
Cellulose	49.7	32.3	2.3	15.6
Hemicellulose	37.4	27.8	7.3	27.4
Cel Hem	45.7	28.6	5.2	20.6
Ara Xyl	22.0	0.3^n.s.^	13.8	63.9
Xyl Glc	16.4	16.1	18.8	48.7
Glc E	41.8	12.4	4.1	41.7
Xyl E	33.0	16.5	1.2^n.s.^	49.2
Ara E	5.1^n.s.^	25.9	9.62E-11^n.s.^	69.0
DW	24.9	45.5	0.7%	28.9
Glc E DW	17.9	60.2	0.5^n.s.^	21.5
Xyl E DW	10.4^n.s.^	54.7	0.1^n.s.^	34.8
Ara E DW	2.0^n.s.^	25.9	0.00E+00^n.s.^	72.1

Variance components are expressed as percentage of the total variance and were estimated using LMEMs in which the effects of genetic groups, genotypes and biological replicates were all treated as random (see Materials and Methods). Superscripts “n.s.” indicate LMEM terms that were not statistically significant at α = 0.05.

A series of variables describing the composition and structural features of the cell wall were collected (**Table S4**). Measurements of acid detergent fibre (ADF), neutral detergent fibre (NDF), acid detergent lignin (ADL), ash, dry matter (DM), and Klason Lignin were determined by near-infrared (NIR) spectroscopy. The amounts of cellulose and hemicellulose were derived as described by Van Soest (1963) and Van Soest et al. (1991). Ratios between the content of certain monosaccharides and cellulose/hemicellulose were also used as indicators of structural cell wall features.

The general trend for these composition variables was that genetic group effects were substantial (i.e., as high as 50% of the total variance for cellulose and 49% for ADF, **Table 2**) and once again mainly driven by contrasts involving *M. floridulus* (**Figure S4**). In contrast to monosaccharides, variation among genotypes within groups also tended to be high (i.e., up to 53% for Klason Lignin) and was only not statistically significant for DM (**Table 2**). Monosaccharide ratios followed a similar trend, though both genetic group and genotype-within-group effects were substantially weaker (**Table 2**; **Figure S5**). As expected, variance components for the cellulose/hemicellulose ratio were roughly intermediate to those for cellulose and hemicellulose (**Table 2**; **Figure S5**).

In summary, genetic group effects for cell wall composition were typically strong but driven almost exclusively by differences between *M. floridulus* (M5) and the other genetic groups. However, there was a comparably high amount of genetic variation among genotypes within groups, indicating that breeding could be effective both within and among groups.

### 3.5 Variation in cell wall recalcitrance

**Figure 4:**
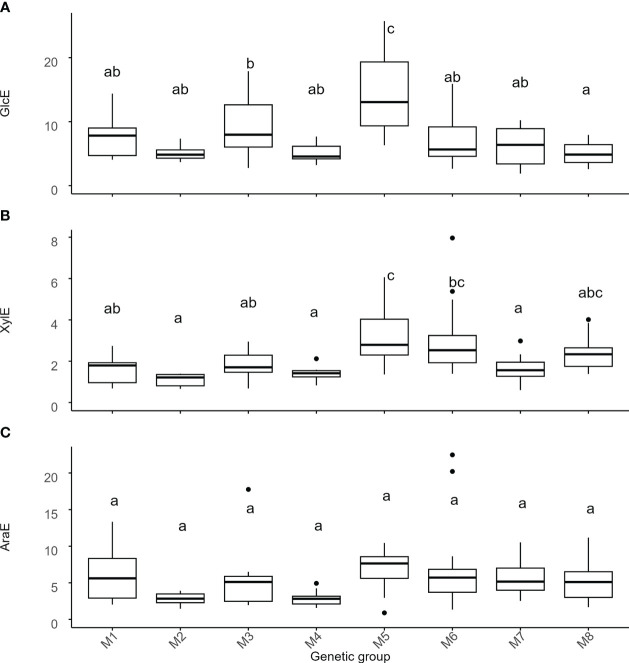
Variation of recalcitrance across genetic groups of *Miscanthus*. Recalcitrance was expressed as the percentage of the total amount of the respective monosaccharides present in the cell wall. Values on the y-axis are the amount of **(A)** glucose (GlcE), **(B)** xylose (XylE), and **(C)** arabinose (AraE) enzymatically released. The thick line in the box represents the median value. The box itself indicates the interquartile range, where 75% of measurements fall. Letters represent significant differences as detected by estimation of marginal means after a LMEM with genetic group treated as a fixed effect and with p < 0.05. Labels on the x-axis are the 8 genetic groups delineated using single-nucleotide polymorphism data (**Table 1**, **Figure 1**). M1 = *M. sinensis* from South Japan, M2 = *M. sinensis* EMI/PRI, M3 = *M. sinensis* from North Japan, M4 = *M. sinensis* from Taiwan, M5 = *M. floridulus*, M6 = *M. sacchariflorus/robustus*, M7 = *M. × giganteus*, M8 = *M. lutarioriparius*.

Next, we wanted to investigate if there are differences in cell wall recalcitrance to sugar release between *Miscanthus* genotypes from different genetic groups. To measure recalcitrance, the cell wall material from aboveground senesced biomass was digested for 48 hours using an enzymatic mixture containing various cellobiohydrolases, and endo-(1 → 4)-β-glucanases with collateral xylanase activity. To increase the ability to detect genetically related differences in recalcitrance, the cell wall material was not pre-treated. The relative recalcitrance was quantified using the amount of glucose (GlcE), xylose (XylE) and arabinose (AraE) released in solution after the digestion and was expressed as a percentage of the total amount of the respective monosaccharides present in the cell wall (**Figure 4**).

Unlike monosaccharide quantities, genetic group effects for GlcE and XylE were strong to moderate (i.e., 42% and 33% of the total variance, respectively, **Tables 2**, **S5**) and once again driven by contrasts involving *M. floridulus*, which was consistently less recalcitrant. This was not the case for AraE, but there was significant variation among genotypes within groups (i.e., 12-26% of the total variance) for all three measures (GlcE, XylE, and AraE) of recalcitrance.

### 3.6 Cell wall recalcitrance in perspective to biomass yield

The amount of monosaccharides that can be enzymatically released from biomass depends not only on cell wall recalcitrance but also on the annual biomass yield. Therefore, we normalized our enzymatic sugar release data (GlcE, XylE and AraE) against the amount of biomass that can be harvested annually from each genotype [i.e., yield in kg of dry weight (DW)].

**Figure 5:**
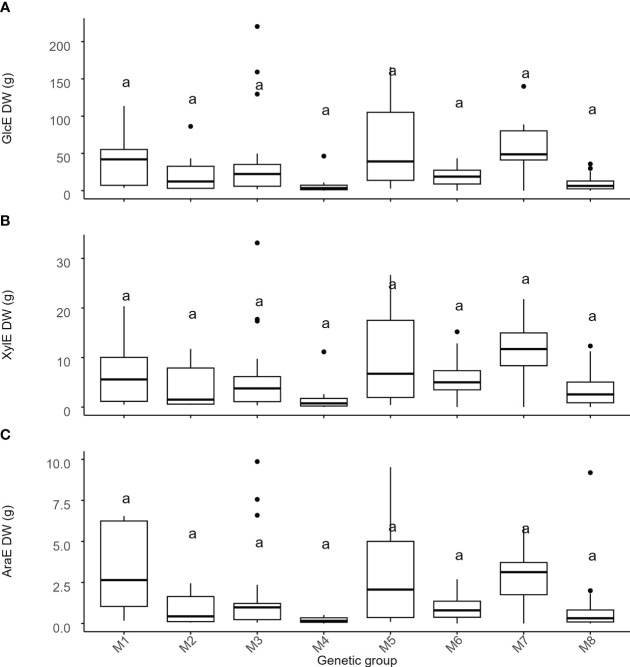
Variation of recalcitrance across genetic groups of *Miscanthus. Miscanthus* biomass to enzymatic sugar release normalized for the amount of biomass produced. Values on the y-axis are the total amount of **(A)** glucose (GlcE), **(B)** xylose (XylE) and **(C)** arabinose (AraE) enzymatically released normalized for the amount of biomass (dry weight) produced. The thick line in the box represents the median value. The box itself indicates the interquartile range, where 75% of measurements fall. Letters represent significant differences as detected by estimation of marginal means after a LMEM with genetic group treated as a fixed effect and with p < 0.05. Labels on the x-axis are the 8 genetic groups identified in the *Miscanthus* population. M1 = *M. sinensis* from South Japan, M2 = *M. sinensis* EMI/PRI, M3 = *M. sinensis* from North Japan, M4 = *M. sinensis* from Taiwan, M5 = *M. floridulus*, M6 = *M. sacchariflorus/robustus*, M7 = *M. × giganteus*, M8 = *M. lutarioriparius*.

As expected, genetic groups differed significantly for DW (**Tables 2**, **S5**), with the *M. × giganteus* group having the highest biomass yields (**Figure S6**). However, these differences accounted for only 25% of the total variance, whereas variation among genotypes within genetic groups was considerably more pronounced (i.e., 46% of the total variance). Consequently, DW-normalized recalcitrance varied strongly among genotypes within groups (i.e., up to 60% of the total variance for DW-normalized GlcE), whereas group effects were weak and only significant for GlcE (**Tables 2**, **S5**), though no specific group contrasts were statistically significant (**Figure 5**). Thus, recalcitrance and biomass yield tended to offset each other at the genetic group level, but with very high levels of genetic variation within groups.

### 3.7 Structural bases of the differences in recalcitrance

**Figure 6:**
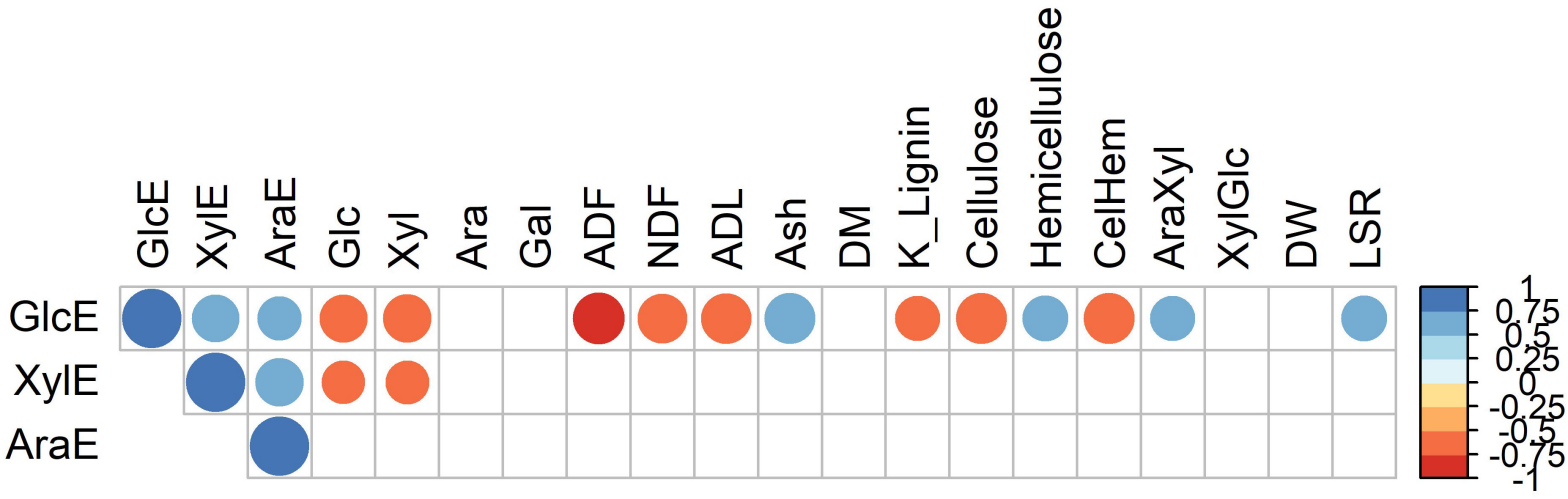
Pearson correlation coefficients between glucose (GlcE), xylose (XylE) and arabinose (AraE) enzymatically released from the cell wall and measures of cell wall structure and composition. Circle sizes are proportional to the significance of the correlation. Only correlations with p < 0.05 (i.e., after Bonferroni corrections) are shown.

We next wanted to determine the compositional and structural cell wall features contributing to the observed differences in recalcitrance to enzymatic sugar release. To explore the structural basis of recalcitrance across the 49 selected *Miscanthus* genotypes, independent from their genetic groups, correlation analysis with Bonferroni correction was performed between enzymatic sugar release (GlcE, XylE, and AraE) and measures for cell wall composition and structure (**Figure 6**). GlcE and XylE were negatively correlated with the total amount of glucose and xylose in the cell wall (r< −0.75), while only GlcE was negatively correlated with lignin content (K lignin and ADL). Furthermore, there was a positive correlation between the ash content and the amount of glucose released. This was also the case for the arabinose-to-xylose ratio. The cellulose-to-hemicellulose ratio negatively correlated with GlcE (**Figure 6**). Finally, there was a positive correlation between the leaf-to-stem ratio (LSR) and GlcE, possibly due to the lower recalcitrance of leaf material when compared to stem material (da Costa et al., 2017). Besides pointing to the complexity of the interactions between compositional and structural cell wall features and cell wall recalcitrance to sugar release, these results also provide some indications of the traits that require attention in breeding programs that target specific end uses of biomass.

### 3.8 Variation in cell wall related variables within and among genetic groups

**Figure 7:**
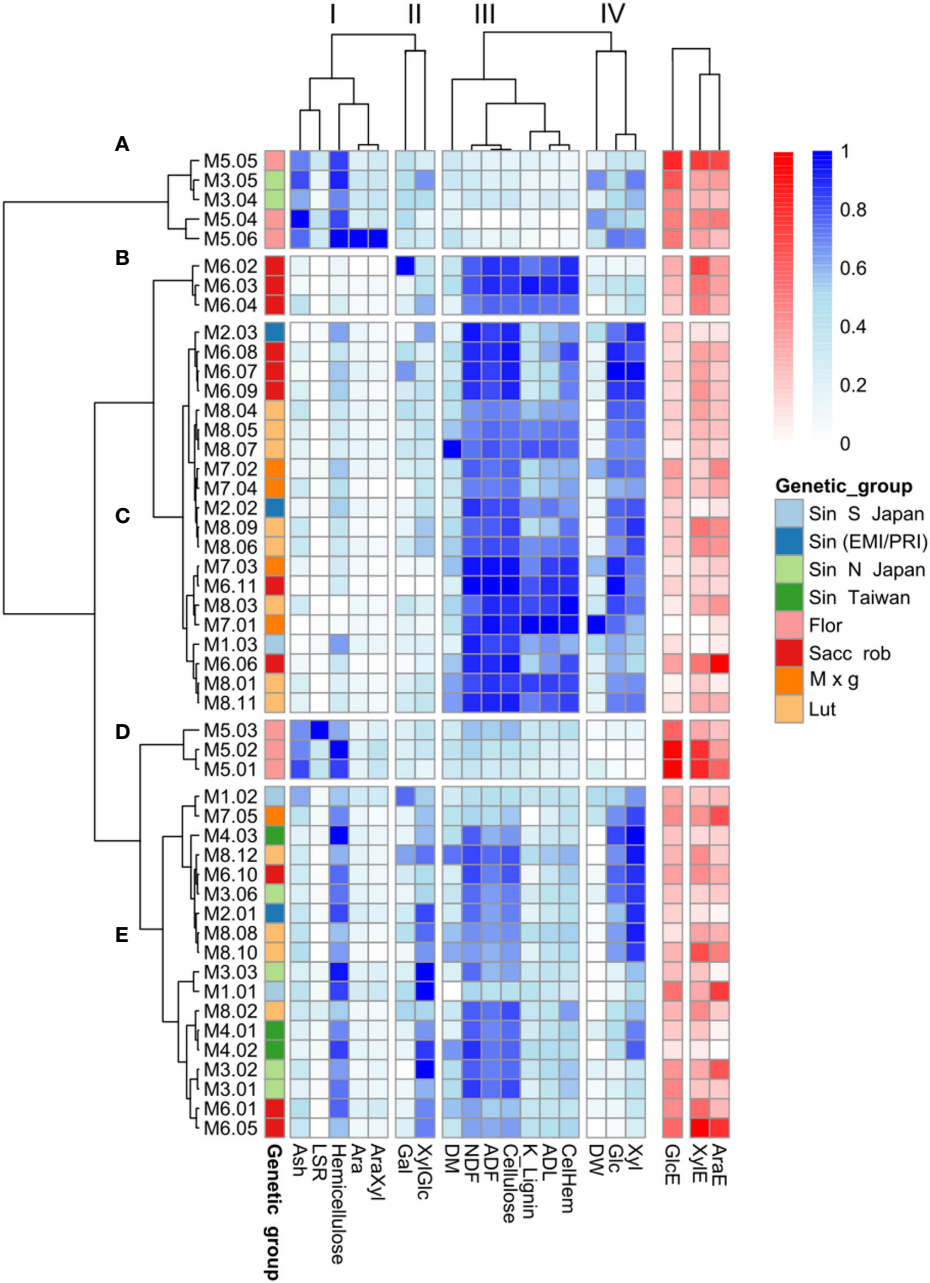
Clustering of the 49 *Miscanthus* genotypes based on cell wall traits. Relative abundances of cell wall compositional features are represented in shades of blue. Shades of red represent saccharification efficiency indices: the percentages of total glucose (GlcE), xylose (XylE) and arabinose (AraE) released upon enzymatic saccharification. Only cell wall composition data (blue), and not the three saccharification-related variables (red), were considered for genotype classification. **(A–E)** Represent the correlation clusters between genotypes, based on the cell wall variables and I-IV indicate the correlation clusters between cell wall features across genotypes. M1 = *M. sinensis* from South Japan, M2 = *M. sinensis* EMI/PRI, M3 = *M. sinensis* from North Japan, M4 = *M. sinensis* from Taiwan, M5 = *M. floridulus*, M6 = *M. sacchariflorus/robustus*, M7 = *M. × giganteus*, M8 = *M. lutarioriparius*.

Having established the overall cell wall features that correlate with enzymatic sugar release across the 49 genotypes, we next wanted to examine if these variables resulted in distinctive correlations with *Miscanthus* genotypes and the genetic group they belong to. Measures for the various cell wall features and enzymatic sugar release data were scaled and two trees were generated using the correlation coefficients between cell wall features and genotypes, respectively (**Figure 7**). Cell wall and phenotypic variables clustered in four distinct groups (I-IV), indicating that the variables within each group tend to follow a similar pattern for a given genotype. For example, cluster I comprised the variables hemicellulose (Hem), arabinose (Ara), arabinose to xylose ratio (AraXyl), leaf to stem ratio (LSR), and ash content (**Figure 7**). These variables (except Ara) all correlated positively with GlcE (**Figure 6**).

Based on the cell wall characteristics (excluding recalcitrance), the genotypes clustered in 5 groups (A-E). The most distinguishing feature of the genotypic tree is that all the *M. floridulus* genotypes (M5) are included in two small clusters (cluster A and cluster D). For both of these clusters, measures of cell wall and phenotypic variables belonging to cluster I tended to be higher for *M. floridulus* genotypes when compared to most other genotypes (**Figure 7**). In addition, both groups A and D contained the genotypes with the lowest recalcitrance (high GlcE values). Most of the genotypes with the highest glucose content (Glc) group together in cluster C, dominated by *M. sacchariflorus/robustus* (M6), *M. × giganteus* (M7), and *M. lutarioriparius* (M8); interestingly this is also the cluster with the highest recalcitrance (low GlcE values).

## 4 Discussion

Despite the potential of *Miscanthus* as a biomass feedstock for the production of fuels and chemicals, few studies have evaluated if biomass recalcitrance to sugar release is governed by the same cell wall features when considering different genetic backgrounds. In this study, we evaluated *Miscanthus* genotypes from eight distinct genetic groups and identified the compositional and structural properties that correlate with the enzymatic release of cell wall sugars. Results suggest that the cell wall properties that correlate with recalcitrance are mostly similar when comparing different genetic *Miscanthus* groups and that differences in biomass allocation to leaf and stem between these groups may contribute to differences in cell wall properties and recalcitrance. Of immediate practical interest, there was significant genetic variation within groups for almost all traits, suggesting that breeding for cell wall characteristics can be effective both within and among groups.

### 4.1 Geospatial analysis to inform the collection of wild accessions

Our analysis of the geographical information of the area of collection of each genotype in the wild showed the opportunity offered by the GBIF database as a source of information for future germplasm collection campaigns. We showed how there are vast areas where the presence of *Miscanthus* has been reported, but have not yet been targeted for the collection of genotypes for breeding programmes. The idea that geographical and environmental information should drive germplasm selection was proposed already in 1972 (Simmonds, 1972). More recently, it was argued that the importance of geographical information for breeders resides in the direct influence of the environmental conditions of an area on the phenotype and genotype of the organisms through adaptation (Parra-Quijano et al., 2012).

In this study, we used a PCA approach to study the contribution of climatic and soil variables to the species’ geographical distribution. A similar approach has been used to study the geographic distribution of tomato accessions (Ramírez-Ojeda et al., 2021) and barley (Teklemariam et al., 2022). The first component of the PCA showed a strong association with the soil pH and total annual precipitation and genotypes of *Miscanthus* under investigation separated along this dimension, with the genotypes of *M. floridulus* forming a separate group. A study using the *Miscanthus* Genomic DataBase (MGDB) and characterizing 485 genotypes of *Miscanthus* (Xiang et al., 2020), showed that 10% of elite germplasm of *M. floridulus* covers most areas in southern China and authors inferred that soil characteristics could play a role. Notably, soil pH is one of the principal determinants of the chemical availability of minerals in the soil (Roem and Berendse, 2000; Stark et al., 2012; Penn and Camberato, 2019) and adaptation to different soil pH conditions could point to differences in nutrient use efficiency and mineral mobilization among *Miscanthus* species and/or genetic groups. It has been shown that the pH of the rhizosphere of *M*. *floridulus* decreases when grown in heavy metal-contaminated soils while the content of soil organic matter increases. Furthermore, the level of heavy metals was low in the rhizosphere compared with the non-rhizosphere, due to the increased uptake of heavy metals (Qin et al., 2022). Knowledge of the level of adaptation to soil pH could inform the choice of breeding specifically for phytoremediation.

### 4.2 Variation in cell wall composition between genetic groups

*M. floridulus* genotypes clearly showed the most distinctive cell wall compositional features. Accessions belonging to this group showed a lower glucose, xylose and ADL content and a higher Ara/Xyl ratio, when compared with some of the other groups. It is known that the composition of leaf and stem cell walls significantly differs in *Miscanthus* (da Costa et al., 2014; da Costa et al., 2017). Glucose, which is mainly derived from cellulose, and lignin content tend to be higher in stems while arabinose and the Ara/Xyl ratio tend to be higher in leaves (da Costa et al., 2017). Thus, the relative proportions of leaf versus stem biomass in the total aboveground biomass influence cell wall compositional features. For example, the leaf-to-stem ratio (LSR) for *M. floridulus* (M5) was significantly higher (LSR = 3.51) compared with those of the other genetic groups (LSR ranging from 0.12 in M8 to 0.70 in M4). Therefore, some of the observed differences in cell wall composition for *M. floridulus* are likely caused by the fact that most of their biomass is comprised of leaf tissue. This is corroborated by the high levels of ash found in *M. floridulus* as it is well established that leaves have a higher ash content than stems (Monti et al., 2008). High levels of ash negatively affect biomass quality for combustion. Another aspect that distinguishes *M. floridulus* from the other genetic groups is that it does not senesce over winter, exhibiting a stay-green phenotype. It has been shown before that genotypes of *M. sinensis* with a higher percentage of the leaf over the total amount of biomass and the ability to keep the leaves over-winter, are characterised by better performance for the production of methane through anaerobic digestion compared to *M.* × *giganteus* (Mangold et al., 2019). From an agronomic point of view, *M. floridulus* could help to increase the harvesting window for *Miscanthus*, when considering their biomass utilization in biorefining and anaerobic digestion.

Effects of genotype and genetic group on cell wall composition have been reported previously. For example, Allison et al. (2011) studied the cell wall composition of 244 genotypes belonging to *M.* × *giganteus*, *M. sacchariflorus*, and *M. sinensis*, and found that *M.* × *giganteus* biomass contained significantly more NDF and cellulose, and less hemicellulose compared with *M. sacchariflorus* or *M. sinensis*. Clustering of eight *Miscanthus* genotypes following a detailed cell wall analysis resulted in two distinctive groupings, a cluster comprised of the *M. sinensis* genotypes and a cluster comprised of hybrids and *M. sacchariflorus* (da Costa et al., 2019). Analysis of 15 accessions of *M. sinensis*, *M. sacchariflorus*, and *M.* × *giganteus* grown at different locations found that differences in cell wall composition between genotypes were mainly caused by genotype-by-environment (G x E) interactions (van der Weijde et al., 2017a).

We found that there were high levels of genetic variation within groups for nearly all cell wall composition traits. This result underlies the existence of a high percentage of intra-specific variation in species of *Miscanthus.* The existence of intra-specific variability within species of Miscanthus had been pointed out by Slavov et al. (2013b). In addition, our results agree with the one presented by Xu et al. (2020) that reported the existence of intra-specific variability in cell wall composition in *M. sinensis*, *M. floridulus*, *M. nudipes*, *M. sacchariflorus*, *M. lutarioriparius*, and their hybrids. Intra-specific genetic variability has been reported for *M. sinensis* (Shimono et al., 2013), *M. lutarioriparius* (Yang et al., 2019), and *M.* × *giganteus* (Głowacka et al., 2015). Thus, breeding programmes aimed at improving cell wall traits do not necessarily need to rely on inter-specific hybrids.

Further research, using growth under controlled environmental stress conditions and multiple genetic groups, is required to determine the full extent of variability in cell wall composition and its plasticity in relation to the environment within the genus *Miscanthus*.

### 4.3 Differences in cell wall recalcitrance between genetic groups

Cell wall recalcitrance is a trait hindering our ability to profitably deconstruct the cell wall to obtain molecules for the biorefining process. In our study, we used the amount of the main cell wall sugars that can be released enzymatically from purified cell walls as a measure of recalcitrance. Effects of genotype on cell wall recalcitrance have been reported before between hybrids and *M. sinensis* (Belmokhtar et al., 2017). Our results show significant differences in cell wall recalcitrance between genetic groups in the genus *Miscanthus*.

It is striking that while genotypes of *M. × giganteus* show the highest content of glucose in the cell wall and *M. floridulus* the lowest, when it comes to recalcitrance the situation is reversed, with *M. floridulus* being the genetic group with the lowest recalcitrance and the highest levels of glucose released. A similar trend can be observed for xylose. However, when the biomass yield is taken into account, the higher recalcitrance of *M. × giganteus* genotypes is offset by their higher yield when compared with *M. floridulus*, resulting in similar amounts of sugars that can be released from *Miscanthus* plants belonging to these two genetic groups. *M. lutarioriparius* showed similar characteristics to *M. × giganteus* for the content and enzymatic release of cell wall sugars, but their lower biomass yield meant that the amount of sugars released on a plant biomass basis remained lower when compared to *M. × giganteus*. Although the abundance of the main cell wall sugars is similar across the four *M. sinensis* groups, genotypes from Japan are less recalcitrant to enzymatic glucose release compared with the other two similar yielding *M. sinensis* groups.

In addition, our results point to the existence of intra-specific variability in cell wall recalcitrance in the *Miscanthus* species under study. Intra-specific variability in recalcitrance has been reported before in biomass crops. For example, Ohlsson et al. (2019) described intra-specific variability in recalcitrance between 286 natural *Salix viminalis* clones. Ostos Garrido et al. (2018), working with different genotypes of three *Poaceae* species, found that the intra-specific variability in recalcitrance is a trait that depends on the species investigated. Our findings emphasize that both biomass yield and biomass recalcitrance need to be taken into account when developing new *Miscanthus* cultivars for biorefining purposes. Moreover, the intra-specific variability available for this trait could be a resource for breeding programs targeting specific biomass end uses.

### 4.4 Structural bases of the differences in cell wall recalcitrance

It has been shown that recalcitrance is a complex trait depending both on the cell wall composition and the structural organization of its components (De Souza et al., 2015). The main components of recalcitrance vary according to the species considered (DeMartini et al., 2013). We investigated the correlation between the cell wall composition and structural features and the amount of glucose, xylose and arabinose enzymatically released to determine which cell wall traits affected recalcitrance. Although da Costa et al. (2019) previously carried out such a correlation analysis, here the group of genotypes used included a wider genetic background as demonstrated by the number of species represented with a large geographical distribution. In addition, the set of cell wall and structural variables used in the current study was significantly different. Not surprisingly, our correlation test confirmed the negative effect of lignin content on the amount of glucose that can be enzymatically released from the cell wall. Lignin represents between 15 and 25% of *Miscanthus* biomass (da Costa et al., 2014), and its content has been shown to play a significant role in recalcitrance (Chen and Dixon, 2007). Indeed, a previous study showed that lignin content correlated negatively with cell wall sugar release in *Miscanthus* (da Costa et al., 2019). However, it has been observed that the monomeric composition of lignin, in particular the syringyl (S) to guaiacyl (G) lignin monomer ratio also has a major role in cell wall recalcitrance (Yoo et al., 2018). Here only the lignin content was used for the correlation and we cannot exclude that a full characterization of the lignin could have provided a better understanding of the relationship observed between the traits. Several studies have shown that lignin is not the only contributor to cell wall recalcitrance (Studer et al., 2011; DeMartini et al., 2013; da Costa et al., 2017; da Costa et al., 2019). DeMartini et al. (2013) compared the cell wall components affecting the recalcitrance of switchgrass and poplar biomass. They found that while lignin removal reduces poplar biomass recalcitrance, xylose removal is more effective in reducing switchgrass biomass recalcitrance. Similarly, Mangold et al. (2019) found that hemicellulose rather than lignin content had a higher effect on methane production from Miscanthus biomass. Indeed, our results show that hemicellulose content and the arabinose to xylose (AraXyl) ratio have a positive correlation with enzymatic glucose release. This is possibly linked to the positive effect of LSR on glucose release as the hemicellulose content and AraXyl ratio tends to be higher in leaves than in stems. Indeed, da Costa et al. (2019) found that the AraXyl ratio correlated positively with glucose release in *Miscanthus* leaves. Similarly, the positive correlation between ash content and sugar release is possibly also linked to LSR. More detailed analysis, evaluating leaf and stem organs separately and expanding the cell wall variables to include for instance lignin composition, measures for pectins and hydroxycinnamic acids could provide further information to explain the differences in cell wall recalcitrance observed.

In summary, we identified a number of cell wall related variables important for biomass quality related to using *Miscanthus* as a biomass crop for biorefining. Although our study identified significant variation in cell wall related features across the 49 selected *Miscanthus* genotypes belonging to eight different genetic groups, with the exception of *M. floridulus*, this variation was generally not distinctive enough to separate the genotypes according to their genetic background. The results emphasize the inter- and intra- specific variation in cell wall characteristics and biomass recalcitrance in the genus *Miscanthus* and the importance of also considering yield and organ related parameters when analyzing cell wall properties and biomass recalcitrance aimed at improving *Miscanthus* as a biomass crop.

## Data availability statement

The original contributions presented in the study are included in the article/**Supplementary Material**. Further inquiries can be directed to the corresponding author.

## Author contributions

RI, GA and MB contributed to conception and design of the study. CD and GS performed the experimental work and data analysis aimed at defining genetic groups. GS provided assistance with the LMEM statistical analyses of cell wall characteristics. JC-B instigated the collection of the NIRS data. All remaining experimental work and data analysis was performed by RI. RI and MB wrote the initial manuscript draft with feedback from GA and GS. All authors contributed to the article and approved the submitted version.

## References

[B1] Albersheim P. Darvil A. Roberts K. Sederof R. Staehelin A. (2011). Plant cell walls: from chemistry to biology (New York: Garland Science (Taylor & Francis Group). doi: 10.1086/662480

[B2] Allison G. G. Morris C. Clifton-Brown J. Lister S. J. Donnison I. S. (2011). Genotypic variation in cell wall composition in a diverse set of 244 accessions of miscanthus. Biomass Bioenergy 35, 4740–4747. doi: 10.1016/j.biombioe.2011.10.008

[B3] Baez L. A. Tichá T. Hamann T. (2022). Cell wall integrity regulation across plant species. Plant Mol. Biol. 109, 483–504. doi: 10.1007/s11103-022-01284-7 35674976 PMC9213367

[B4] Barnes W. J. Anderson C. T. (2017). Acetyl bromide soluble lignin (ABSL) assay for total lignin quantification from plant biomass. Bio-Protocol 7, e2149. doi: 10.21769/BioProtoc.2149 34458465 PMC8376531

[B5] Barnes W. J. Anderson C. T. (2018). Release, recycle, rebuild: cell-wall remodeling, autodegradation, and sugar salvage for new wall biosynthesis during plant development. Mol. Plant 11, 31–46. doi: 10.1016/j.molp.2017.08.011 28859907

[B6] Bates D. Mächler M. Bolker B. Walker S. (2015). Fitting linear mixed-effects models using lme4. J. Stat. Soft. 67, 1–48. doi: 10.18637/jss.v067.i01

[B7] Belmokhtar N. Arnoult S. Chabbert B. Charpentier J.-P. Brancourt-Hulmel M. (2017). Saccharification performances of miscanthus at the pilot and miniaturized assay scales: genotype and year variabilities according to the biomass composition. Front. Plant Sci. 8, 740. doi: 10.3389/fpls.2017.00740 28611790 PMC5447034

[B8] Bilska-Kos A. Pietrusińska A. Suski S. Niedziela A. Linkiewicz A. M. Majtkowski W.. (2022). Cell wall properties determine genotype-specific response to cold in *Miscanthus* × *giganteus* plants. Cells 11, 547. doi: 10.3390/cells11030547 35159356 PMC8834381

[B9] Brancourt-Hulmel M. Arnoult S. Cézard L. El Hage F. Gineau E. Girones J.. (2022). A comparative study of maize and miscanthus regarding cell-wall composition and stem anatomy for conversion into bioethanol and polymer composites. Bioenerg. Res. 15, 777–791. doi: 10.1007/s12155-020-10239-z

[B10] Chen F. Dixon R. A. (2007). Lignin modification improves fermentable sugar yields for biofuel production. Nat. Biotechnol. 25, 759–761. doi: 10.1038/nbt1316 17572667

[B11] Clifton-Brown J. Harfouche A. Casler M. D. Dylan Jones H. Macalpine W. J. Murphy-Bokern D.. (2019a). Breeding progress and preparedness for mass-scale deployment of perennial lignocellulosic biomass crops switchgrass, miscanthus, willow and poplar. GCB Bioenergy 11, 118–151. doi: 10.1111/gcbb.12566 30854028 PMC6392185

[B12] Clifton-Brown J. Schwarz K. U. Awty-Carroll D. Iurato A. Meyer H. Greef J.. (2019b). Breeding strategies to improve miscanthus as a sustainable source of biomass for bioenergy and biorenewable products. Agronomy 9, 673. doi: 10.3390/agronomy9110673

[B13] da Costa R. Allison G. Bosch M. (2015). Cell wall biomass preparation and Fourier transform mid-infrared (FTIR) spectroscopy to study cell wall composition. Bio-Protocol 5 (11), e1494. doi: 10.21769/BioProtoc.1494

[B14] da Costa R. M. F. Lee S. J. Allison G. G. Hazen S. P. Winters A. Bosch M. (2014). Genotype, development and tissue-derived variation of cell-wall properties in the lignocellulosic energy crop miscanthus. Ann. Bot. 114, 1265–1277. doi: 10.1093/aob/mcu054 24737720 PMC4195551

[B15] da Costa R. M. F. Pattathil S. Avci U. Lee S. J. Hazen S. P. Winters A.. (2017). A cell wall reference profile for miscanthus bioenergy crops highlights compositional and structural variations associated with development and organ origin. New Phytol. 213, 1710–1725. doi: 10.1111/nph.14306 27859277 PMC5324610

[B16] da Costa R. M. F. Pattathil S. Avci U. Winters A. Hahn M. G. Bosch M. (2019). Desirable plant cell wall traits for higher-quality miscanthus lignocellulosic biomass. Biotechnol. Biofuels 12, 1–18. doi: 10.1186/s13068-019-1426-7 31011368 PMC6463665

[B17] DeMartini J. D. Pattathil S. Miller J. S. Li H. Hahn M. G. Wyman C. E. (2013). Investigating plant cell wall components that affect biomass recalcitrance in poplar and switchgrass. Energy Environ. Sci. 6, 898–909. doi: 10.1039/C3EE23801F

[B18] De Souza A. P. Kamei C. L. A. A. Torres A. F. Pattathil S. Hahn M. G. Trindade L. M.. (2015). How cell wall complexity influences saccharification efficiency in *Miscanthus sinensis* . J. Exp. Bot. 66, 4351–4365. doi: 10.1093/jxb/erv183 25908240 PMC4493786

[B19] De Vega J. Donnison I. Dyer S. Farrar K. (2021). Draft genome assembly of the biofuel grass crop *Miscanthus sacchariflorus* . F1000Res 10, 29. doi: 10.12688/f1000research.44714.1 33732433 PMC7921889

[B20] Domon J. M. Baldwin L. Acket S. Caudeville E. Arnoult S. Zub H.. (2013). Cell wall compositional modifications of miscanthus ecotypes in response to cold acclimation. Phytochemistry 85, 51–61. doi: 10.1016/j.phytochem.2012.09.001 23079767

[B21] Fao/Iiasa/Isric/Isscas/Jrc (2009). Harmonized world soil database (version 1.1) (Rome, Italy and IIASA, Laxenburg, Austria: FAO).

[B22] Fick S. E. Hijmans R. J. (2017). WorldClim 2: new 1-km spatial resolution climate surfaces for global land areas. Int. J. Climatology 37, 4302–4315. doi: 10.1002/joc.5086

[B23] Foster C. E. Martin T. M. Pauly M. (2010). Comprehensive compositional analysis of plant cell walls (lignocellulosic biomass) part I: lignin. J. Visualized Experiments 37, e1745. doi: 10.3791/1837 PMC3144576 20224547

[B24] Fry S. C. (1988). “The growing plant cell wall: chemical and metabolic analysis,” in Longman scientific & technical (Caldwell: The Blackburn Press). Available at: https://books.google.co.uk/books?id=WITwAAAAMAAJ.

[B25] Gbif.Org (2019). Occurrence download. 454461. doi: 10.15468/DL.KTFBBB

[B26] Gladala-Kostarz A. Doonan J. H. Bosch M. (2020). Mechanical stimulation in *Brachypodium distachyon*: implications for fitness, productivity, and cell wall properties. Plant Cell Environ. 43, 1314–1330. doi: 10.1111/pce.13724 31955437 PMC7318644

[B27] Głowacka K. Clark L. V. Adhikari S. Peng J. Stewart J. R. Nishiwaki A.. (2015). Genetic variation in *Miscanthus × giganteus* and the importance of estimating genetic distance thresholds for differentiating clones. GCB Bioenergy 7, 386–404. doi: 10.1111/gcbb.12166

[B28] Hijmans R. J. Cameron S. E. Parra J. L. Jones P. G. Jarvis A. (2005). Very high resolution interpolated climate surfaces for global land areas. Int. J. Climatology 25, 1965–1978. doi: 10.1002/joc.1276

[B29] Hijmans R. J. Elith J. (2013). Species distribution modeling with R. R Cran Project.

[B30] Hijmans R. J. Spooner D. M. (2001). Geographic distribution of wild potato species. Am. J. Bot. 88, 2101–2112. doi: 10.2307/3558435 21669641

[B31] Hoang N. V. Furtado A. Botha F. C. Simmons B. A. Henry R. J. (2015). Potential for genetic improvement of sugarcane as a source of biomass for biofuels. Front. Bioeng. Biotechnol. 3, 182. doi: 10.3389/fbioe.2015.00182 26636072 PMC4646955

[B32] Hodkinson T. R. Renvoize S. (2001). Nomenclature of *Miscanthus* x *giganteus* (*Poaceae*). Kew Bull. 56, 759. doi: 10.2307/4117709

[B33] Hothorn T. Bretz F. Westfall P. (2008). Simultaneous inference in general parametric models. Biometrical J. 50, 346–363. doi: 10.1002/bimj.200810425 18481363

[B34] Huang L. S. Flavell R. Donnison I. S. Chiang Y.-C. Hastings A. Hayes C.. (2019). Collecting wild miscanthus germplasm in Asia for crop improvement and conservation in Europe whilst adhering to the guidelines of the united nations’ convention on biological diversity. Ann. Bot. 124, 591–604. doi: 10.1093/aob/mcy231 30596965 PMC6821356

[B35] Hyman G. Hodson D. Jones P. (2013). Spatial analysis to support geographic targeting of genotypes to environments. Front. Physiol. 4, 40. doi: 10.3389/fphys.2013.00040 23515351 PMC3600773

[B36] Jombart T. (2008). Adegenet: a r package for the multivariate analysis of genetic markers. Bioinformatics 24, 1403–1405. doi: 10.1093/bioinformatics/btn129 18397895

[B37] Jombart T. Devillard S. Balloux F. Falush D. Stephens M. Pritchard J.. (2010). Discriminant analysis of principal components: a new method for the analysis of genetically structured populations. BMC Genet. 11, 94. doi: 10.1186/1471-2156-11-94 20950446 PMC2973851

[B38] Kalinina O. Nunn C. Sanderson R. Hastings A. F. S. van der Weijde T. Özgüven M.. (2017). Extending miscanthus cultivation with novel germplasm at six contrasting sites. Front. Plant Sci. 8, 563. doi: 10.3389/fpls.2017.00563 28469627 PMC5395641

[B39] Kang S. Post W. M. Nichols J. A. Wang D. West T. O. Bandaru V.. (2013). Marginal lands: concept, assessment and management. J. Agric. Sci. 5, 129–139. doi: 10.5539/jas.v5n5p129

[B40] Kuznetsova A. Brockhoff P. B. Christensen R. H. B. (2017). lmerTest package: tests in linear mixed effects models. J. Stat. Software 82, 1–26. doi: 10.18637/jss.v082.i13

[B41] Le Gall H. Philippe F. Domon J.-M. Gillet F. Pelloux J. Rayon C. (2015). Cell wall metabolism in response to abiotic stress. Plants 4, 112–166. doi: 10.3390/plants4010112 27135320 PMC4844334

[B42] Lenth R. V. (2022) Emmeans: estimated marginal means, aka least-squares means. Available at: https://CRAN.R-project.org/package=emmeans.

[B43] Lewandowski I. Clifton-Brown J. Trindade L. M. van der Linden G. C. Schwarz K.-U. Müller-Sämann K.. (2016). Progress on optimizing miscanthus biomass production for the European bioeconomy: results of the EU FP7 project OPTIMISC. Front. Plant Sci. 7, 1620. doi: 10.3389/fpls.2016.01620 27917177 PMC5114296

[B44] Li X. Liao H. Fan C. Hu H. Li Y. Li J.. (2016). Distinct geographical distribution of the miscanthus accessions with varied biomass enzymatic saccharification. PLoS One 11, e0160026. doi: 10.1371/journal.pone.0160026 27532636 PMC4988763

[B45] Lu F. Lipka A. E. Glaubitz J. Elshire R. Cherney J. H. Casler M. D.. (2013). Switchgrass genomic diversity, ploidy, and evolution: novel insights from a network-based SNP discovery protocol. PLoS Genet. 9 (1), e1003215. doi: 10.1371/journal.pgen.1003215 23349638 PMC3547862

[B46] Lygin A. V. Upton J. Dohleman F. G. Juvik J. Zabotina O. A. Widholm J. M.. (2011). Composition of cell wall phenolics and polysaccharides of the potential bioenergy crop miscanthus. GCB Bioenergy 3, 333–345. doi: 10.1111/j.1757-1707.2011.01091.x

[B47] Maji A. T. Shaibu A. A. (2012). Application of principal component analysis for rice germplasm characterization and evaluation. J. Plant Breed. Crop Sci. 4, 87–93. doi: 10.5897/JPBCS11.093

[B48] Malinowska M. Donnison I. S. Robson P. R. H. (2017). Phenomics analysis of drought responses in miscanthus collected from different geographical locations. GCB Bioenergy 9, 78–91. doi: 10.1111/gcbb.12350

[B49] Mangold A. Lewandowski I. Möhring J. Clifton-Brown J. Krzyżak J. Mos M.. (2019). Harvest date and leaf:stem ratio determine methane hectare yield of miscanthus biomass. GCB Bioenergy 11, 21–33. doi: 10.1111/gcbb.12549

[B50] McCann M. C. Carpita N. C. (2015). Biomass recalcitrance : a multi-scale , multi-factor , and conversion-specific property. J. Exp. Bot. 66, 4109–4118. doi: 10.1093/jxb/erv267 26060266

[B51] Mitros T. Session A. M. James B. T. Wu G. A. Belaffif M. B. Clark L. V.. (2020). Genome biology of the paleotetraploid perennial biomass crop miscanthus. Nat. Commun. 11, 5442. doi: 10.1038/s41467-020-18923-6 33116128 PMC7595124

[B52] Monti A. Di Virgilio N. Venturi G. (2008). Mineral composition and ash content of six major energy crops. Biomass Bioenergy 32, 216–223. doi: 10.1016/j.biombioe.2007.09.012

[B53] Moura J. C. M. S. Bonine C. A. V. de Oliveira Fernandes Viana J. Dornelas M. C. Mazzafera P. (2010). Abiotic and biotic stresses and changes in the lignin content and composition in plants. J. Integr. Plant Biol. 52, 360–376. doi: 10.1111/j.1744-7909.2010.00892.x 20377698

[B54] Ohlsson J. A. Hallingbäck H. R. Jebrane M. Harman-Ware A. E. Shollenberger T. Decker S. R.. (2019). Genetic variation of biomass recalcitrance in a natural salix viminalis (L.) population. Biotechnol. Biofuels 12, 135. doi: 10.1186/s13068-019-1479-7 31171936 PMC6545741

[B55] Oshunsanya S. O. Aliku O. (2016). “GIS applications in agronomy,” in Geospatial technology - environmental and social applications. Eds. Imperatore P. Pepe A. (London, UK: InTech). doi: 10.5772/64528

[B56] Ostos Garrido F. J. Pistón F. Gómez L. D. McQueen-Mason S. J. (2018). Biomass recalcitrance in barley, wheat and triticale straw: correlation of biomass quality with classic agronomical traits. PLoS One 13, e0205880. doi: 10.1371/journal.pone.0205880 30403701 PMC6221549

[B57] Oyedeji O. Langholtz M. Hellwinckel C. Webb E. (2021). Supply analysis of preferential market incentive for energy crops. Biofuels Bioprod. Bioref. 15, 736–748. doi: 10.1002/bbb.2184

[B58] Pancaldi F. Trindade L. M. (2020). Marginal lands to grow novel bio-based crops: a plant breeding perspective Front. Plant Sci. 11, 227. doi: 10.3389/fpls.2020.00227 32194604 PMC7062921

[B59] Parra-Quijano M. Iriondo J. M. Torres E. (2012). Review. applications of ecogeography and geographic information systems in conservation and utilization of plant genetic resources. Span J. Agric. Res. 10, 419. doi: 10.5424/sjar/2012102-303-11

[B60] Penn C. Camberato J. (2019). A critical review on soil chemical processes that control how soil pH affects phosphorus availability to plants. Agriculture 9, 120. doi: 10.3390/agriculture9060120

[B61] Petit J. Gulisano A. Dechesne A. Trindade L. M. (2019). Phenotypic variation of cell wall composition and stem morphology in hemp (*Cannabis sativa* l.): optimization of methods. Front. Plant Sci. 10, 959. doi: 10.3389/fpls.2019.00959 31402925 PMC6671528

[B62] Pettolino F. A. Walsh C. Fincher G. B. Bacic A. (2012). Determining the polysaccharide composition of plant cell walls. Nat. Protoc. 7, 1590–1606. doi: 10.1038/nprot.2012.081 22864200

[B63] Qin J. Zhao H. Dai M. Zhao P. Chen X. Liu H.. (2022). Speciation distribution and influencing factors of heavy metals in rhizosphere soil of *Miscanthus floridulus* in the tailing reservoir area of dabaoshan iron polymetallic mine in northern guangdong. Processes 10, 1217. doi: 10.3390/pr10061217

[B64] Ramírez-Ojeda G. Peralta I. E. Rodríguez-Guzmán E. Chávez-Servia J. L. Sahagún-Castellanos J. Rodríguez-Pérez J. E. (2021). Climatic diversity and ecological descriptors of wild tomato species (*Solanum* sect. *Lycopersicon*) and close related species (*Solanum* sect. *Juglandifolia* and sect. *Lycopersicoides*) in Latin America. Plants 10, 855. doi: 10.3390/plants10050855 33922706 PMC8145878

[B65] Rancour D. M. Marita J. M. Hatfield R. D. (2012). Cell wall composition throughout development for the model grass *Brachypodium distachyon* . Front. Plant Sci. 3, 266. doi: 10.3389/fpls.2012.00266 23227028 PMC3514619

[B66] R Core Team. (2018) R: a language and environment for statistical computing. Available at: https://www.r-project.org/.

[B67] Resch M. G. Baker J. O. Decker S. R. (2015). Low solids enzymatic saccharification of lignocellulosic biomass. Tech. Rep. NREL/TP-5100-63351 Lab. Analytical Procedure (LAP), 1–9.

[B68] Roem W. J. Berendse F. (2000). Soil acidity and nutrient supply ratio as possible factors determining changes in plant species diversity in grassland and heathland communities. Biol. Conserv. 92, 151–161. doi: 10.1016/S0006-3207(99)00049-X

[B69] Saeman J. F. (1945). Kinetics of wood saccharification - hydrolysis of cellulose and decomposition of sugars in dilute acid at high temperature. Ind. Eng. Chem. 37, 43–52. doi: 10.1021/ie50421a009

[B70] Santoro N. Cantu S. L. Tornqvist C. E. Falbel T. G. Bolivar J. L. Patterson S. E.. (2010). A high-throughput platform for screening milligram quantities of plant biomass for lignocellulose digestibility. Bioenergy Res. 3, 93–102. doi: 10.1007/s12155-009-9074-6

[B71] Shimono Y. Kurokawa S. Nishida T. Ikeda H. Futagami N. (2013). Phylogeography based on intraspecific sequence variation in chloroplast DNA of *Miscanthus sinensis* (Poaceae), a native pioneer grass in Japan. Botany 91, 449–456. doi: 10.1139/cjb-2012-0212

[B72] Simmonds N. W. (1972). Genetic resources in plants - their exploration and conservation. edited by o. h. Frankel and e. Bennett Oxford: Blackwell scientific publications (1970). Ex. Agric. 8, 87–87. doi: 10.1017/S0014479700023553

[B73] Slavov G. Allison G. Bosch M. (2013b). Advances in the genetic dissection of plant cell walls: tools and resources available in miscanthus. Front. Plant Sci. 4, 217. doi: 10.3389/fpls.2013.00217 23847628 PMC3701120

[B74] Slavov G. T. Nipper R. Robson P. Farrar K. Allison G. G. Bosch M.. (2014). Genome-wide association studies and prediction of 17 traits related to phenology, biomass and cell wall composition in the energy grass *Miscanthus sinensis* . New Phytol. 201, 1227–1239. doi: 10.1111/nph.12621 24308815 PMC4284002

[B75] Slavov G. Robson P. Jensen E. Hodgson E. Farrar K. Allison G.. (2013a). Contrasting geographic patterns of genetic variation for molecular markers vs. phenotypic traits in the energy grass *Miscanthus sinensis* . GCB Bioenergy 5, 562–571. doi: 10.1111/gcbb.12025

[B76] Sluiter A. Hames B. Ruiz R. Scarlata C. Sluiter J. Templeton D.. (2008). Determination of structural carbohydrates and lignin in biomass. Lab. Anal. Proced. 1617 (1), 1–16.

[B77] Stark S. Eskelinen A. Männistö M. K. (2012). Regulation of microbial community composition and activity by soil nutrient availability, soil pH, and herbivory in the tundra. Ecosystems 15, 18–33. doi: 10.1007/s10021-011-9491-1

[B78] Studer M. H. DeMartini J. D. Davis M. F. Sykes R. W. Davison B. Keller M.. (2011). Lignin content in natural populus variants affects sugar release. Proc. Natl. Acad. Sci. 108, 6300–6305. doi: 10.1073/pnas.1009252108 21444820 PMC3076829

[B79] Tamura K. Uwatoko N. Yamashita H. Fujimori M. Akiyama Y. Shoji A.. (2016). Discovery of natural interspecific hybrids between *Miscanthus sacchariflorus* and *Miscanthus sinensis* in southern Japan: morphological characterization, genetic structure, and origin. Bioenergy Res. 9, 315–325. doi: 10.1007/s12155-015-9683-1

[B80] Tang C. Yang X. Chen X. Ameen A. Xie G. (2018). Sorghum biomass and quality and soil nitrogen balance response to nitrogen rate on semiarid marginal land. Field Crops Res. 215, 12–22. doi: 10.1016/j.fcr.2017.09.031

[B81] Teklemariam S. S. Bayissa K. N. Matros A. Pillen K. Ordon F. Wehner G. (2022). The genetic diversity of Ethiopian barley genotypes in relation to their geographical origin. PLoS One 17, e0260422. doi: 10.1371/journal.pone.0260422 35622864 PMC9140232

[B82] The Plant List. (2013). The plant list. version 1.1. Available at: http://www.theplantlist.org/.

[B83] Torres A. F. Xu X. Nikiforidis C. Bitter J. H. Trinidade L. M. (2019). Exploring the treasure of plant molecules with integrated biorefineries. Front. Plant. Sci. 10, 478. doi: 10.3389/fpls.2019.00478 31040858 PMC6476976

[B84] Vaahtera L. Schulz J. Hamann T. (2019). Cell wall integrity maintenance during plant development and interaction with the environment. Nat. Plants 5, 924–932. doi: 10.1038/s41477-019-0502-0 31506641

[B85] van der Weijde T. Dolstra O. Visser R. G. F. Trindade L. M. (2017a). Stability of cell wall composition and saccharification efficiency in miscanthus across diverse environments. Front. Plant Sci. 7, 2004. doi: 10.3389/fpls.2016.02004 28111583 PMC5216675

[B86] van der Weijde T. Kamei C. L. A. Severing E. I. Torres A. F. Gomez L. D. Dolstra O.. (2017b). Genetic complexity of miscanthus cell wall composition and biomass quality for biofuels. BMC Genomics 18, 1–15. doi: 10.1186/s12864-017-3802-7 28545405 PMC5445440

[B87] Van Soest P. J. (1963). Use of detergents in the analysis of fibrous feeds. 2. a rapid method for the determination of fiber and lignin. J. Assoc. Off. Agric. Chem. 46, 829–835. doi: 10.1093/jaoac/46.5.829

[B88] Van Soest P. J. Robertson J. B. Lewis B. A. (1991). Methods for dietary fiber, neutral detergent fiber, and nonstarch polysaccharides in relation to animal nutrition. J. Dairy Sci. 74, 3583–3597. doi: 10.3168/jds.S0022-0302(91)78551-2 1660498

[B89] Von Cossel M. Lewandowski I. Elbersen B. Staritsky I. Van Eupen M. Iqbal Y.. (2019). Marginal agricultural land low-input systems for biomass production. Energies 12, 3123. doi: 10.3390/en12163123

[B90] Wieczorek J. Bloom D. Guralnick R. Blum S. Döring M. Giovanni R.. (2012). Darwin Core: an evolving community-developed biodiversity data standard. PLoS One 7 (1), e29715. doi: 10.1371/journal.pone.0029715 22238640 PMC3253084

[B91] Xiang W. Xue S. Liu F. Qin S. Xiao L. Yi Z. (2020). MGDB: a database for evaluating *Miscanthus* spp. to screen elite germplasm. Biomass Bioenergy 138, 105599. doi: 10.1016/j.biombioe.2020.105599

[B92] Xu P. Cheng S. Han Y. Zhao D. Li H. Wang Y.. (2020). Natural variation of lignocellulosic components in miscanthus biomass in China. Front. Chem. 8, 595143. doi: 10.3389/fchem.2020.595143 33251186 PMC7674668

[B93] Yang Y. Reilly E. C. Jungers J. M. Chen J. Smith T. M. (2019). Climate benefits of increasing plant diversity in perennial bioenergy crops. One Earth 1, 434–445. doi: 10.1016/j.oneear.2019.11.011

[B94] Yang S. Xue S. Kang W. Qian Z. Yi Z. (2019). Genetic diversity and population structure of miscanthus lutarioriparius, an endemic plant of China. PLoS One 14, e0211471. doi: 10.1371/journal.pone.0211471 30707722 PMC6358086

[B95] Yoo C. G. Dumitrache A. Muchero W. Natzke J. Akinosho H. Li M.. (2018). Significance of lignin S/G ratio in biomass recalcitrance of *Populus trichocarpa* variants for bioethanol production. ACS Sustain. Chem. Eng. 6, 2162–2168. doi: 10.1021/acssuschemeng.7b03586

[B96] Zhang G. Ge C. Xu P. Wang S. Cheng S. Han Y.. (2021). The reference genome of *Miscanthus floridulus* illuminates the evolution of *Saccharinae* . Nat. Plants 7, 608–618. doi: 10.1038/s41477-021-00908-y 33958777 PMC8238680

