# Supplementary material for: Variability of cell wall recalcitrance and composition in genotypes of Miscanthus from different genetic groups and geographical origin
Source: Front Plant Sci. 2023 Jun 6;14:1155188. doi: 10.3389/fpls.2023.1155188 (PMC10279889; doi:10.3389/fpls.2023.1155188)
Supplement: Supplementary file 1 [file DataSheet_1.pdf]

Supplementary Material

Variability of cell wall recalcitrance and composition in genotypes of *Miscanthus* from different genetic groups and geographical origin

Rosario Iacono, Gancho T. Slavov, Christopher L. Davey, John Clifton-Brown, Gordon Allison, Maurice Bosch*

*** Correspondence: mub@aber.ac.uk**

1.1 Supplementary Figures

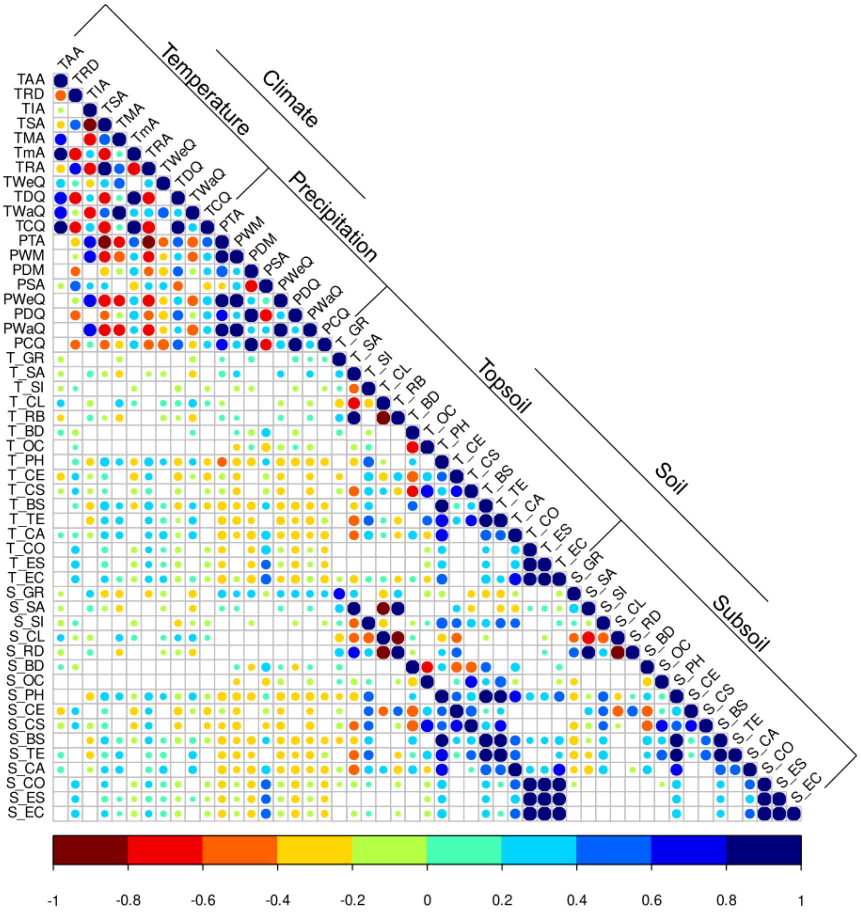

Figure S1. Pearson correlation coefficients of source soil and climate variables for the 592 genotypes in the ABR33 trial. Circle sizes are proportional to the significance of the correlation. Correlations with p > 0.01 (i.e., after Bonferroni corrections) are not shown (white spaces). Variable descriptions are available in Table S1 and Table S2.

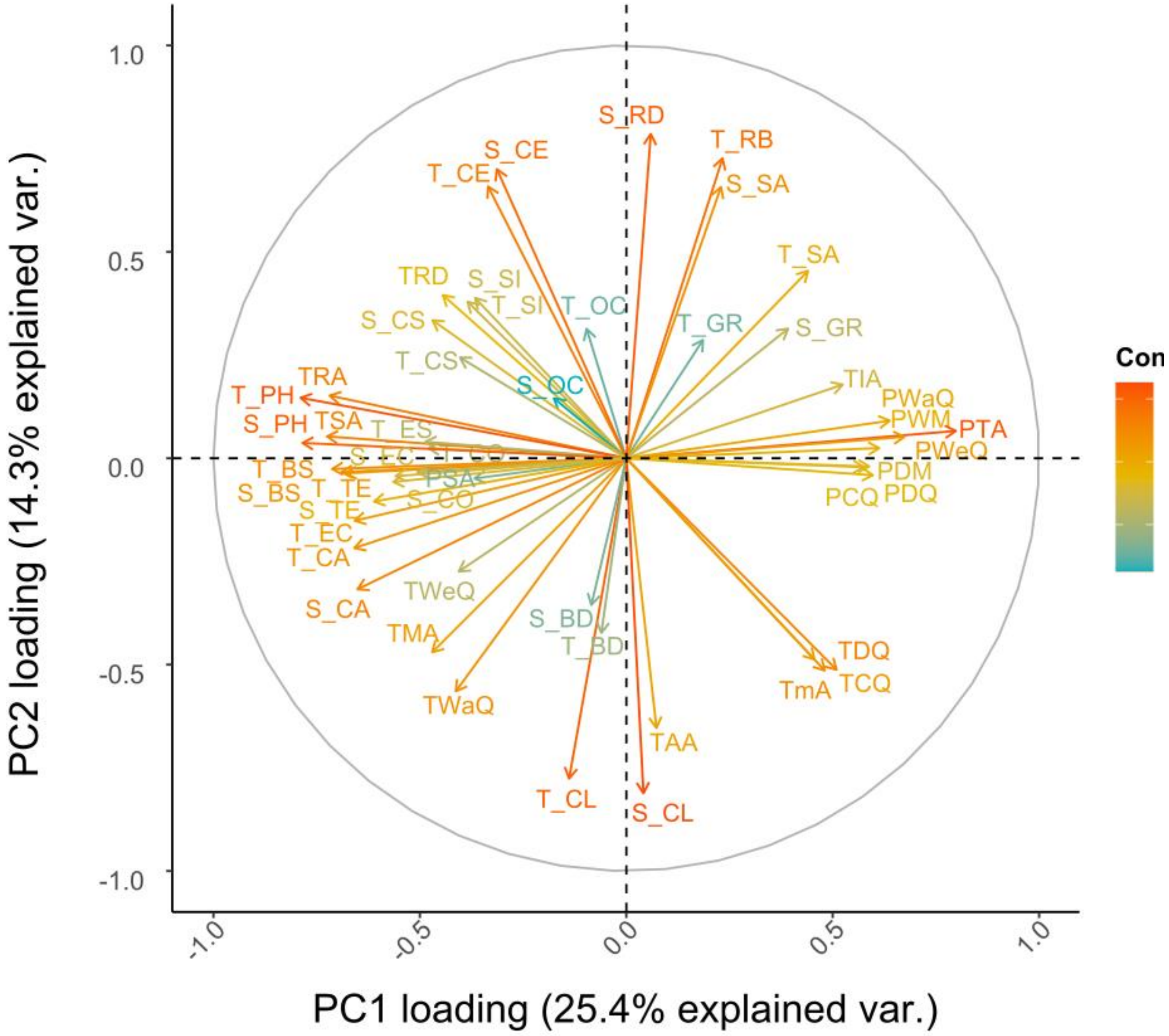

Figure S2. Principal component analysis (PCA) of climate and soil variables for sampling locations of ABR33 genotypes. Loadings for the first and second principal components (PC1 and PC2) are shown along the x- and y-axis, respectively with proportions of variance explained shown in parenthesis. Labels indicate climate and soil variables (the full list and description of the variables are available in Table S1 and Table S2). The size and color scale associated with the arrows indicates the contribution of each variable to the components.

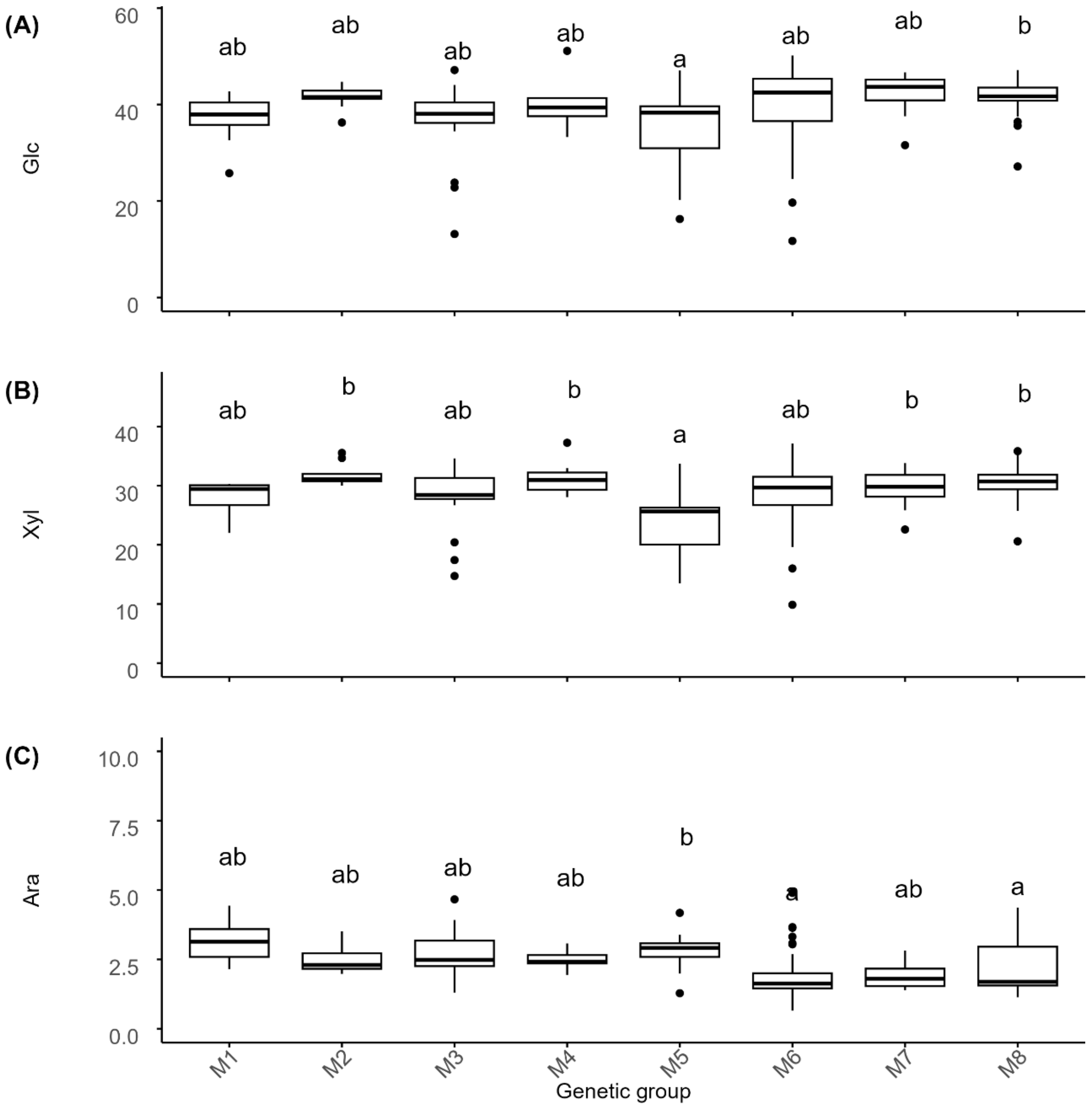

Figure S3. Effect of the genetic group on the amount of monosaccharides released after acid hydrolysis. Values on the y-axes are the amount of (A) glucose (Glc), (B) xylose (Xyl), and (C) arabinose (Ara) released as a percentage of the cell wall material (CWM). The thick line in the box represents the median value. The box itself indicates the interquartile range, where 75% of measurements fall. Letters represent significant differences as detected by estimation of marginal means after a LMEM with genetic group treated as a fixed effect and with $p < 0.05$. Labels on the x-axes are the 8 genetic *Miscanthus* groups delineated using single-nucleotide polymorphism data (Table 1, Figure 1). M1 = *M. sinensis* from South Japan, M2 = *M. sinensis* EMI/PRI, M3 = *M. sinensis* from North Japan, M4 = *M. sinensis* from Taiwan, M5 = *M. floridulus*, M6 = *M. sacchariflorus/robustus*, M7 = *M.* × *giganteus*, M8 = *M. lutarioriparius.*

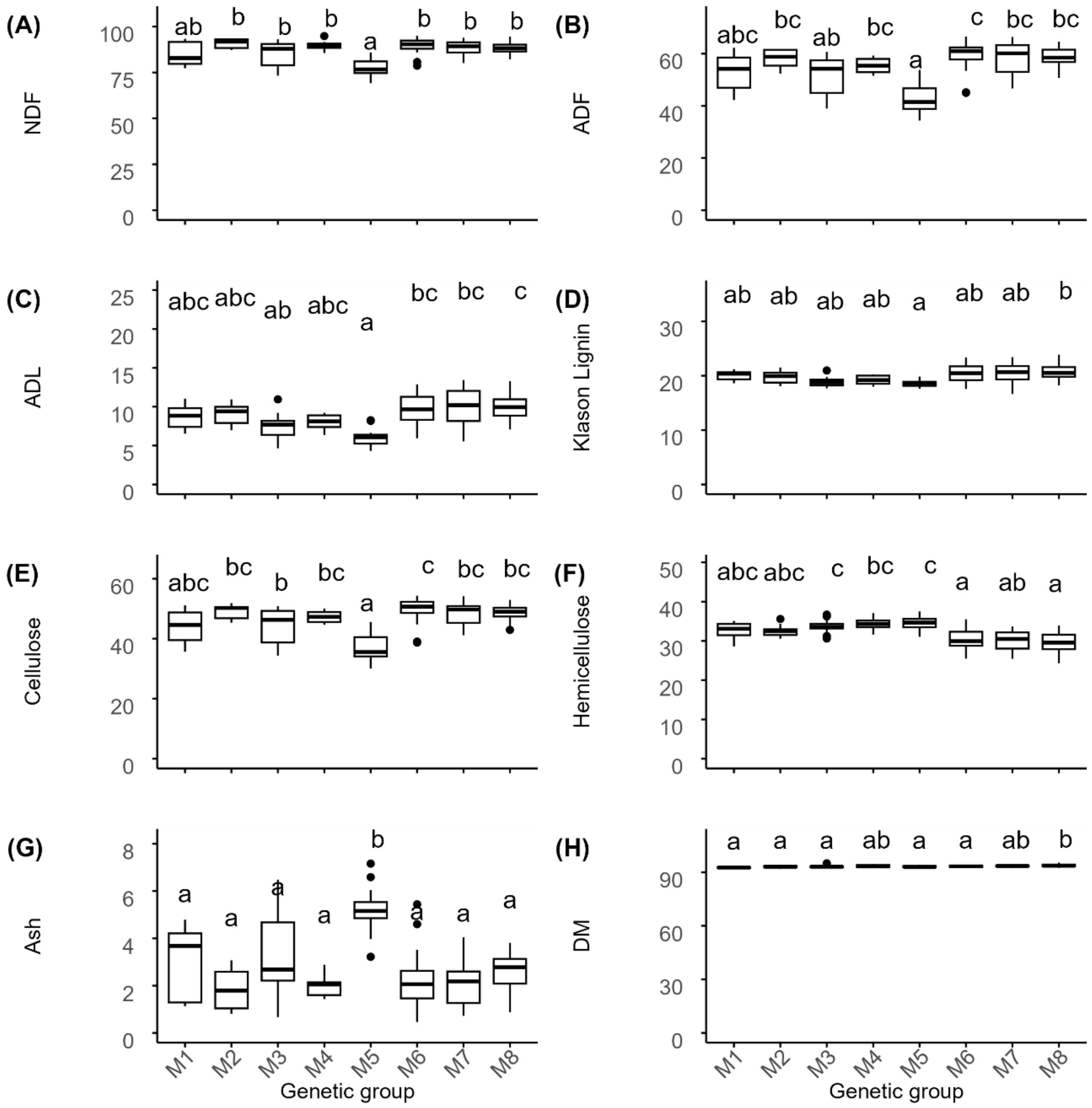

Figure S4. Effect of the genetic group on the cell wall composition as determined by NIRS. Values on the y-axis are the amount of (A) Neutral Detergent Fiber (NDF), (B) Acid Detergent Fiber (ADF), (C) Acid Detergent Lignin (ADL), (D) Klason Lignin, (E) Cellulose, (F) Hemicellulose, (G) Ash expressed as a percentage of the dry matter (DM) and (H) dry matter expressed as a percentage of the fresh weight (FW). The thick line in the box represents the median value. The box itself indicates the interquartile range, where 75% of measurements fall. Letters represent significant differences as detected by estimation of marginal means after a LMEM with genetic group treated as a fixed effect and with $p < 0.05$. Labels on the x-axis are the 8 genetic groups delineated using single-nucleotide polymorphism data (Table 1, Figure 1). M1 = *M. sinensis* from South Japan, M2 = *M. sinensis* EMI/PRI, M3 = *M. sinensis* from North Japan, M4 = *M. sinensis* from Taiwan, M5 = *M. floridulus*, M6 = *M. sacchariflorus/robustus*, M7 = *M. × giganteus*, M8 = *M. lutarioriparius.*

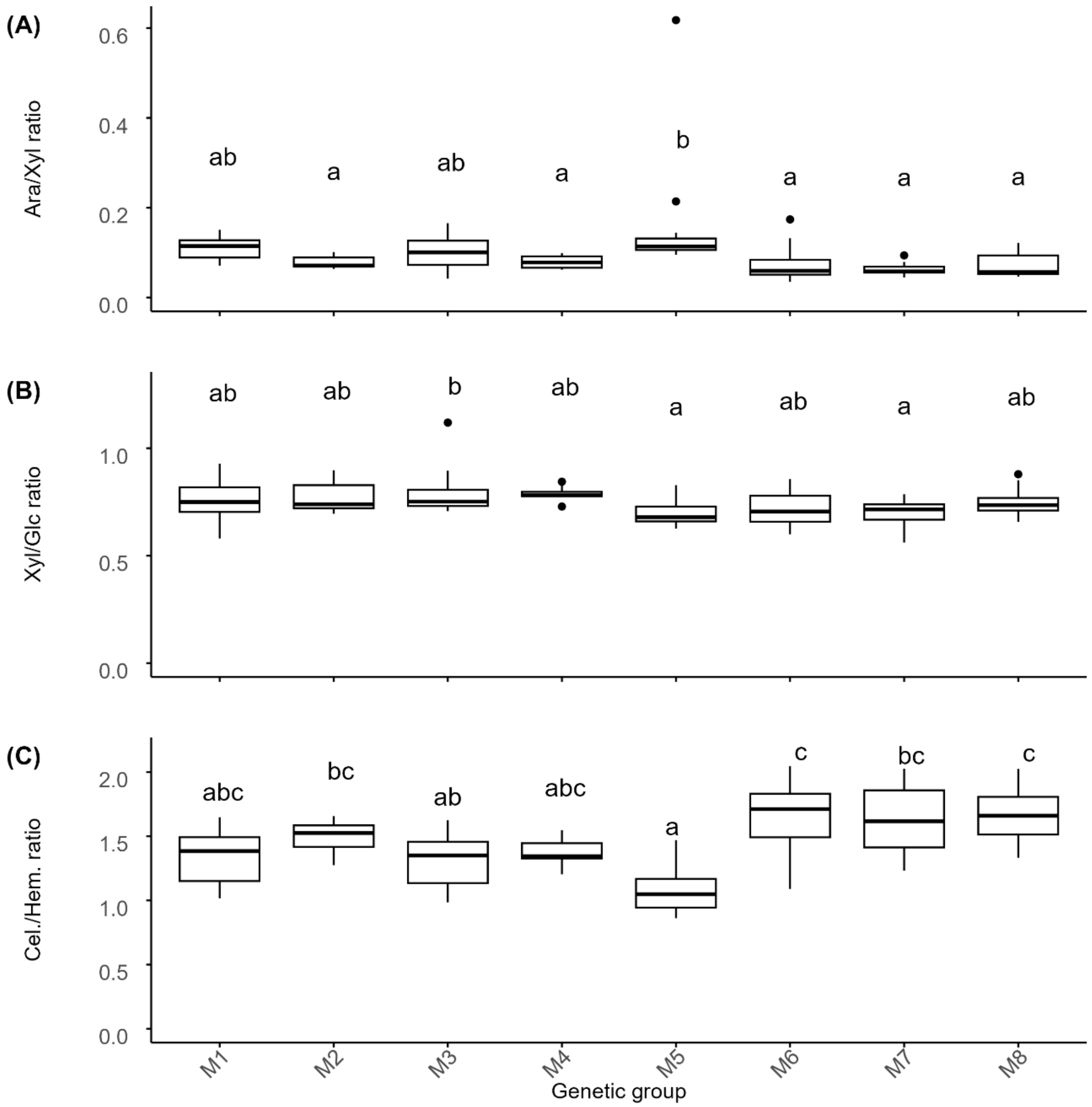

Figure S5. Effect of the genetic group on the cell wall architecture as represented by the ratios between some of the main components. Values on the y-axis are (A) The ratio between Arabinose (Ara) and Xylose (Xyl) (AraXyl), (B) The ratio between Xylose (Xyl) and Glucose (Glc) (XylGlc) and (C) The ratio between Cellulose (Cel) and Hemicellulose (Hem) (CelHem). The thick line in the box represents the median value. The box itself indicates the interquartile range, where 75% of measurements fall. Letters represent significant differences as detected by estimation of marginal means after a LMEM with genetic group treated as a fixed effect and with $p < 0.05$. Labels on the x-axis are the 8 genetic groups delineated using single-nucleotide polymorphism data (Table 1, Figure 1). M1 = *M. sinensis* from South Japan, M2 = *M. sinensis* EMI/PRI, M3 = *M. sinensis* from North Japan, M4 = *M. sinensis* from Taiwan, M5 = *M. floridulus*, M6 = *M. sacchariflorus/robustus*, M7 = *M.* × *giganteus,* M8 = *M. lutarioriparius.*

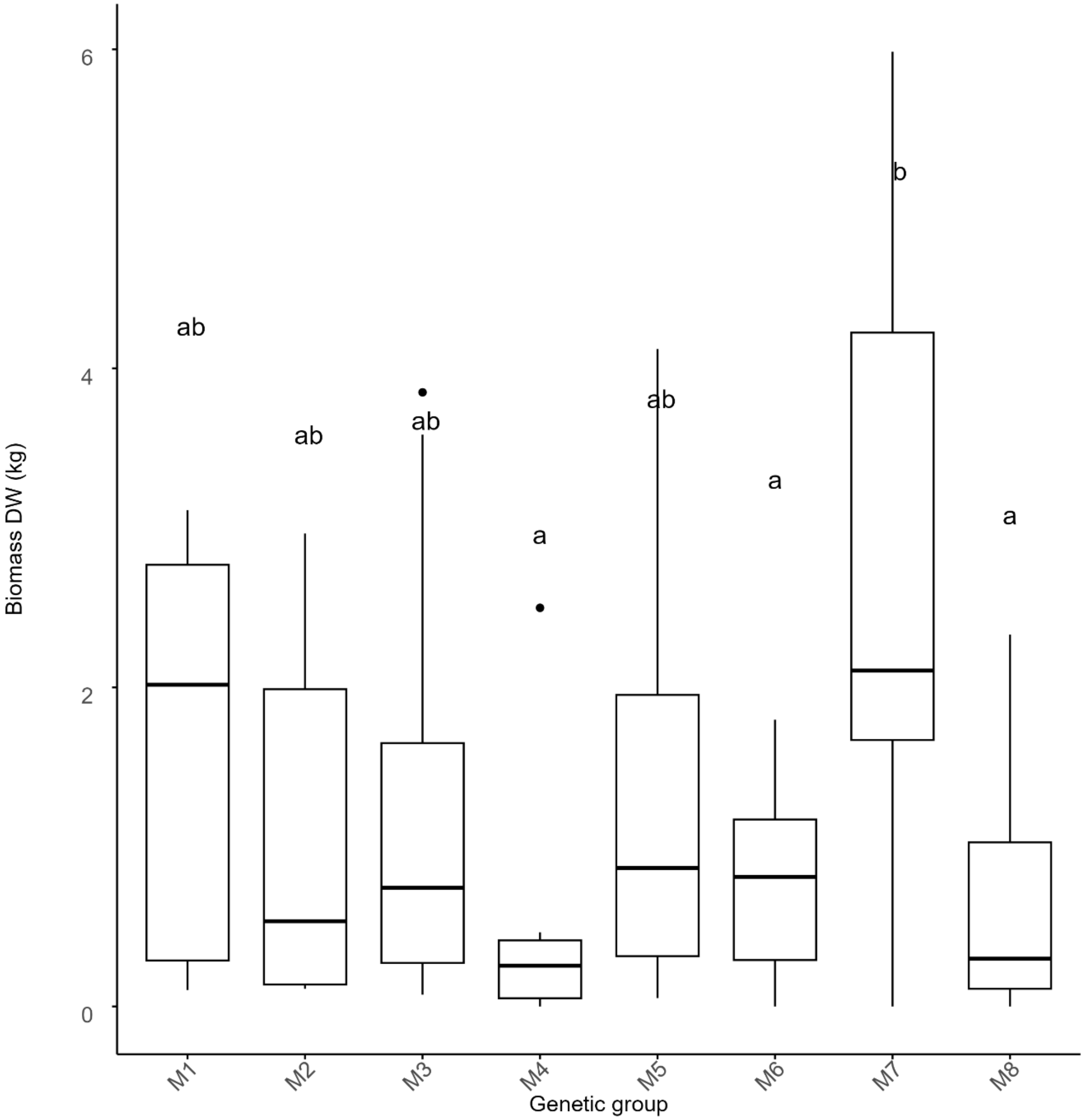

Figure S6. Effect of the genetic group on the amount of biomass yield. Values on the y-axis are the amount of biomass harvested expressed in kilograms of dry weight. The thick line in the box represents the median value. The box itself indicates the interquartile range, where 75% of measurements fall. Letters represent significant differences as detected by estimation of marginal means after a LMEM with genetic group treated as a fixed effect and with $p < 0.05$. Labels on the x-axis are the 8 genetic groups delineated using single-nucleotide polymorphism data (Table 1, Figure 1). M1 = *M. sinensis* from South Japan, M2 = *M. sinensis* EMI/PRI, M3 = *M. sinensis* from North Japan, M4 = *M. sinensis* from Taiwan, M5 = *M. floridulus*, M6 = *M. sacchariflorus/robustus*, M7 = *M.* × *giganteus*, M8 = *M. lutarioriparius*.

1.2 Supplementary Tables

Table S1. List of climate variables used. List of the variables that were used in this analysis to describe precipitation, temperature, and their variation from data collected in the last 50 years. Each variable is identified by an abbreviation (ID) used in figures.

ID	Unit	Description	mean	sd
TAA	°C	Annual average temperature	13.645	3.134
TRD	°C	The annual average of daily range of temperature variation[1]	8.741	1.353
TIA	%	Isothermality. The highest value of the percentage of the annual temperature variation perceived in a day.[2]	28.208	6.187
TSA	%	Coefficient of variation of TAA	8034.247	1802.351
TMA	°C	Maximum annual temperature	29.220	3.708
TmA	°C	Minimal annual temperature	-2.185	5.116
TRA	°C	Annual range of variation of temperature	31.460	5.842
TWeQ	°C	The average temperature in the wettest quarter of the year	21.607	4.127
TDQ	°C	The average temperature in the driest quarter of the year	4.602	5.125
TWaQ	°C	The average temperature in the warmest quarter of the year	23.743	3.445
TCQ	°C	The average temperature in the coldest quarter of the year	3.230	4.415
PTA	mm	Total annual precipitation	1606.760	624.151
PWM	mm	Total precipitation in the wettest month	258.345	101.965
PDM	mm	Total precipitation in the driest month	52.367	28.597
PSA	mm	Annual seasonality of precipitation. Coefficient of variation of PTA.	52.816	20.036
PWeQ	mm	Total precipitation in the wettest quarter	676.485	279.982
PDQ	mm	Total precipitation in the driest quarter	180.693	90.185
PWaQ	mm	Total precipitation in the warmest quarter	626.515	262.690
PCQ	mm	Total precipitation in the coldest quarter	201.856	124.587

[1] Mean Diurnal Range (Mean of monthly (max temp - min temp))

[2] (TRD/TRA)*100

Table S2. List of the soil variables retrieved from the Harmonised World Soil Database (HWSD)

List of the variables that were used in this analysis to describe the physical and chemical characteristics of each soil map unit. The same set of variables was obtained for the topsoil and subsoil layers. Topsoil is represented by the first 100 cm of the soil profile. Subsoil describes the soil below 100 cm of the soil profile. Variables from the topsoil have an ID starting with T and variables from the subsoil start with S. Each variable is identified by an abbreviation (ID) used in figures.

ID	Unit	Description	mean	sd
T_GR		Topsoil gravel content	8.478	3.738
T_SA		Topsoil sand content	36.873	7.732
T_SI		Topsoil silt content	36.609	6.138
T_CL		Topsoil clay content	26.553	7.527
T_RB	kg/dm^3	Topsoil reference bulk density	1.371	0.051
T_BD	kg/dm^3	Topsoil bulk density	1.221	0.158
T_OC		Topsoil organic carbon content	2.470	1.983
T_PH	$-log(H^+)$	pH in the water extract of the topsoil	6.109	0.868
T_CE	cmol/kg	Topsoil cation exchange capacity (CEC) in the clay fraction	45.492	17.129
T_CS	cmol/kg	Topsoil cation exchange capacity (CEC)	17.651	6.508
T_BS		Topsoil base saturation	64.453	22.894
T_TE	cmol/kg	Topsoil total exchangeable bases	11.837	6.565
T_CA		Calcium carbonate content in the topsoil	1.359	3.209
T_CO		Calcium sulfate (gypsum) in the topsoil	0.036	0.254
T_ES		Topsoil sodicity	2.059	3.773
T_EC	dS/m	Topsoil salinity	0.180	0.319
S_GR		Subsoil gravel content	9.786	4.969
S_SA		Subsoil sand content	37.084	7.621
S_SI		Subsoil silt content	33.673	5.786
S_CL		Subsoil clay content	29.268	8.944
S_RD	kg/dm^3	Subsoil reference bulk density	1.358	0.056
S_BD	kg/dm^3	Subsoil bulk density	1.267	0.171
S_OC		Subsoil organic carbon content	1.194	1.448
S_PH	$-log(H^+)$	pH in the water extract of the topsoil	6.185	0.886

ID	Unit	Description	mean	sd
S_CE	cmol/kg	Subsoil cation exchange capacity (CEC) in the clay fraction	49.301	20.461
S_CS	cmol/kg	Subsoil cation exchange capacity (CEC)	16.135	5.861
S_BS		Subsoil base saturation	61.584	26.541
S_TE	cmol/kg	Subsoil total exchangeable bases	11.293	8.756
S_CA		Calcium carbonate content in the topsoil	1.474	3.991
S_CO		Calcium sulfate (gypsum) in the topsoil	0.026	0.144
S_ES		Subsoil sodicity	2.105	3.778
S_EC	dS/m	Subsoil salinity	0.393	1.601

Table S3. Source locations and genetic group membership of the 49 selected genotypes selected for cell wall characterization.

Group ID	pop_dapc8	gen_n	Lat	Long	Alt	Country
M1	Sin_S_Japan	11	40° 54 ' 36"	124° 51 ' 7"	450	China
M1	Sin_S_Japan	37	35° 25 ' 49"	133° 17 ' 2"	2	Japan
M1	Sin_S_Japan	39	35° 17 ' 18"	132° 41 ' 10"	62	Japan
M2	Sin (EMI/PRI)	16	NA	NA	NA	NA
M2	Sin (EMI/PRI)	45	NA	NA	NA	NA
M2	Sin (EMI/PRI)	47	NA	NA	NA	NA
M3	Sin_N_Japan	13	36° 8 ' 28"	137° 43 ' 30"	1320	Japan
M3	Sin_N_Japan	14	37° 0 ' 58"	140° 7 ' 16"	1260	Japan
M3	Sin_N_Japan	15	NA	NA	NA	NA
M3	Sin_N_Japan	19	35° 30 ' 34"	137° 32 ' 35"	460	Japan
M3	Sin_N_Japan	38	35° 36 ' 59"	135° 58 ' 26"	20	Japan
M3	Sin_N_Japan	46	NA	NA	NA	NA
M4	Sin_Taiwan	17	NA	NA	NA	NA
M4	Sin_Taiwan	18	NA	NA	NA	NA
M4	Sin_Taiwan	42	24° 7 ' 14"	121° 16 ' 8"	2997	Taiwan
M5	Flor	1	23° 17 ' 24"	120° 29 ' 53"	2282	Taiwan
M5	Flor	3	24° 3 ' 38"	121° 9 ' 19"	1932	Taiwan
M5	Flor	8	24° 37 ' 31"	121° 29 ' 6"	1213	Taiwan
M5	Flor	9	24° 3 ' 40"	121° 6 ' 58"	2309	Taiwan
M5	Flor	10	23° 29 ' 15"	120° 53 ' 24"	2623	Taiwan
M5	Flor	43	25° 8 ' 36"	121° 31 ' 14"	NA	Taiwan
M6	Sacc_rob	2	24° 11 ' 10"	121° 20 ' 5"	2340	Taiwan
M6	Sacc_rob	4	34° 18 ' 16"	117° 57 ' 4"	12	China
M6	Sacc_rob	5	34° 18 ' 21"	117° 57 ' 4"	12	China
M6	Sacc_rob	6	34° 18 ' 5"	117° 56 ' 42"	18	China
M6	Sacc_rob	7	34° 18 ' 17"	117° 57 ' 4"	24	China
M6	Sacc_rob	12	33° 56 ' 27"	133° 38 ' 6"	222	Japan
M6	Sacc_rob	32	37° 24 ' 41"	118° 39 ' 25"	-1	China
M6	Sacc_rob	33	37° 24 ' 41"	118° 39 ' 25"	-1	China
M6	Sacc_rob	34	37° 24 ' 41"	118° 39 ' 25"	-1	China
M6	Sacc_rob	36	34° 17 ' 56"	117° 57 ' 7"	27	China
M6	Sacc_rob	41	35° 43 ' 60"	127° 34 ' 59"	350	Korea, South
M7	Hyb	20	29° 2 ' 39"	111° 24 ' 54"	36	China
M7	Hyb	40	37° 58 ' 0"	128° 28 ' 1"	510	Korea, South
M7	Hyb	44	NA	NA	NA	NA
M7	Hyb	48	NA	NA	NA	NA
M7	Hyb	49	NA	NA	NA	NA
M8	Lut	21	28° 32 ' 1"	112° 4 ' 37"	33	China
M8	Lut	22	28° 30 ' 45"	112° 14 ' 53"	19	China
M8	Lut	23	28° 32 ' 27"	112° 13 ' 26"	30	China
M8	Lut	24	28° 32 ' 37"	112° 4 ' 30"	23	China

M8	Lut	25	28° 33 ' 8"	112° 5 ' 31"	31	China
M8	Lut	26	28° 49 ' 50"	112° 23 ' 46"	33	China
M8	Lut	27	28° 52 ' 57"	112° 26 ' 20"	30	China
M8	Lut	28	29° 25 ' 42"	113° 3 ' 58"	22	China
M8	Lut	29	28° 35 ' 39"	112° 0 ' 54"	22	China
M8	Lut	30	31° 10 ' 9"	118° 13 ' 16"	2	China
M8	Lut	31	28° 53 ' 9"	112° 6 ' 7"	26	China
M8	Lut	35	29° 1 ' 55"	111° 20 ' 42"	40	China

Table S4. Chemical composition and bioconversion characteristics analysed.

	Trait	Unit	Reference	Description	mean	sd
Composition	Ara	%	CWM	Arabinose content in the cell wall material from the plant biomass from the, determined as D-arabinose by HPAEC-PAD	2.46	1.81
	Gal	%	CWM	Galactose content in the cell wall material from the plant biomass, determined as D-galactose by HPAEC	0.80	0.63
	Glc	%	CWM	Glucose content in the cell wall material from the plant biomass, determined as D-glucose by HPAEC	39.30	7.03
	Xyl	%	CWM	Xylose content in the cell wall material from the plant biomass, determined as D-xylose by HPAEC	28.75	4.81
	NDF	%	DM	Amount of Neutral Detergent Fiber predicted using NIR. Calibration was carried using the method from (Van Soest, 1963).	86.97	5.69
	ADF	%	DM	Amount of Acid Detergent Fiber predicted using NIR. Calibration was carried using the method from (Van Soest, 1963).	55.38	7.40
	ADL	%	DM	Amount of Acid Detergent Lignin predicted using NIR. Calibration was carried using the method from (Van Soest, 1963).	8.86	2.13
	Cellulose	%	DM	Amount of cellulose, calculated according to the formula: $Cellulose = ADF - ADL$	46.51	5.58
	Hemicellulose	%	DM	Amount of hemicellulose calculated according to the formula: $Hemicellulose = NDF - ADF$	31.60	2.96
	K_Lignin	%	DM	Amount of lignin predicted from NIR. Calibration was carried using the method by (Van Soest, 1963; Van Soest et al., 1991)	19.94	1.53
	Ash	%	DM	Amount of ash predicted using NIR. Calibration was carried using the method from (Van Soest, 1963)	2.80	1.42

	Trait	Unit	Reference	Description	mean	sd
	DM	%	Fresh weight	Dry matter content, determined as described in (Huang et al., 2019).	93.30	0.66
Cell Wall Structure	AraXyl			Degree of arabinoxylans substitution expressed as the ratio between Xylose and Arabinose.	0.09	0.06
	XylGlc			Glucose to xylose ratio	0.74	0.07
	CelHem			The ratio between cellulose and hemicellulose	1.50	0.29
Saccharification efficiency	AraE	%		Amount of arabinose released by a certain amount of cell wall upon a 48 digestion with an enzymatic mix containing cellulase and β-glucosidase, expressed as a percentage of D-arabinose over Ara.	5.50	3.32
	GlcE	%		Amount of glucose released by a certain amount of cell wall upon a 48 digestion with an enzymatic mix containing cellulase and β-glucosidase expressed as a percentage of D-glucose over Glc.	7.43	4.52
	XylE	%		Amount of xylose released by a certain amount of cell wall upon a 48 digestion with an enzymatic mix containing cellulase and β-glucosidase expressed as a percentage of D-xylose over Xyl.	2.23	1.11
	AraE_DW	%		Amount of arabinose released by a certain amount of cell wall upon a 48 digestion with an enzymatic mix containing cellulase and β-glucosidase, normalised for the amount of dry biomass produced (DW).	1.98	3.25
	GlcE_DW	%		Amount of glucose released by a certain amount of cell wall upon a 48 digestion with an enzymatic mix containing cellulase and β-glucosidase, normalised for the amount of dry biomass produced (DW).	29.74	39.23
	XylE_DW	%		Amount of xylose released by a certain amount of cell wall upon a 48 digestion with an enzymatic mix containing cellulase and β-	6.00	6.33

	Trait	Unit	Reference	Description	mean	sd
				glucosidase normalised for the amount of dry biomass produced (DW).		
Plant Architecture	DW	g		The dry weight of the material harvested from a plant during the harvesting campaign in 2017. Values are the average of the values for the 3 biological replicates.	1.10	1.19
	DWL	g		The dry weight of leaf material, average of three stems.	11.10	11.29
	DWS	g		The dry weight of stem material, average of three stems.	43.62	51.86
	LSR	%		Leaf to stem ratio expressed as the percentage of the leaf on the stem.	0.80	1.52

1.3 Data and data analysis

The datasets and the code used for the statistical analysis presented in this paper are available at: https://github.com/RosarioIacono/Iaconoetal2023Data

Table S5. Details of the components associated with genetic group (σ^2_{Group}), genotypes within groups (σ^2_{Geno}), biological replicates (σ^2_{Rep}) and residual error (σ^2_{Err}) estimated using LMEMs. In the models, the effects of genetic groups, genotypes and biological replicates were all treated as random (see Materials and Methods). Where the effect was not significant ($p > 0.05$), “n.s.” is indicated next to the values.

	Predictors			Random Effects						
	(Intercept)			Group		Geno		Rep		Err
	Estimates	CI	p	σ^2	p	σ^2	p	σ^2	p	σ^2
**Glc**	39.1	34.71 – 43.49	<0.001	5.20500	0.044	7.357	0.032	11.5470000	4.91E-07	30.148
**Xyl**	28.79	26.20 – 31.38	<0.001	5.40500	0.001	1.69 n.s.	0.275	2.5790000	0.001	15.417
**Ara**	2.6	1.79 – 3.42	<0.001	0.30020	0.044	0.1122 n.s.	0.661	0.3128000	0.019	2.7076
**NDF**	86.81	83.72 – 89.90	<0.001	16.17520	1.99E-05	12.3773	3.53E-14	0.12220 n.s.	0.480	6.6643
**ADF**	54.63	50.41 – 58.85	<0.001	28.27000	1.07E-06	18.4830	1.32E-14	1.4000000	0.002	9.581
**ADL**	8.63	7.55 – 9.72	<0.001	1.71522	9.20E-05	1.82522	3.81E-13	0.0913200	0.031	1.08195
**Ash**	2.78	1.98 – 3.59	<0.001	1.07057	2.10E-06	0.55775	3.31E-08	0.0366400	0.070	0.5655
**DM**	93.21	92.93 – 93.49	<0.001	0.08961	1.99E-04	0.01886 n.s.	0.537	0.01672 n.s.	0.121	0.32465
**K Lignin**	19.8	19.16 – 20.45	<0.001	0.44611	0.021	1.27128	2.80E-15	0.0435400	0.057	0.62685
**Cellulose**	46.01	42.80 – 49.21	<0.001	16.45850	1.23E-06	10.70000	2.09E-15	0.7594000	0.002	5.1761
**Hemicellulose**	32.17	30.46 – 33.87	<0.001	3.45350	1.01E-05	2.56770	1.94E-08	0.6759000	2.33E-05	2.5319
**Cel Hem**	1.45	1.28 – 1.62	<0.001	0.03899	6.16E-07	0.02443	3.65E-11	0.0044040	4.29E-05	0.017579
**Ara Xyl**	0.09	0.06 – 0.12	<0.001	7.52E-04	4.93E-05	1.15E-05 n.s.	0.954	0.0004703	0.001	2.18E-03
**Xyl Glc**	0.74	0.70 – 0.79	<0.001	9.98E-04	0.012	9.79E-04	0.007	0.0011402	5.80E-07	0.0029609
**Glc E**	7.55	5.03 – 10.06	<0.001	9.31980	4.67E-07	2.75490	0.014	0.9106000	0.019	9.2968
**Xyl E**	2.03	1.50 – 2.56	<0.001	0.44225	1.39E-04	0.22064	0.007	0.01633 n.s.	0.384	0.65911
**Ara E**	5.44	4.55 – 6.33	<0.001	5.68E-01 n.s.	0.515	2.89100	0.006	1.07E-09 n.s.	1.000	7.71E+00
**DW**	1.22	0.69 – 1.75	<0.001	0.37499	0.011	0.68626	0.000	0.0112700	0.358	0.43588
**Glc E DW**	32.95	17.14 – 48.76	<0.001	284.91300	0.040	958.23900	< 2e-16	7.28100 n.s.	0.436	341.623
**Xyl E DW**	6.23	4.08 – 8.38	<0.001	4.2754 n.s.	0.204	22.56810	4.71E-12	0.02310 n.s.	0.941	14.3686
**Ara E DW**	2.06	1.32 – 2.81	<0.001	0.2125 n.s.	0.680	2.75420	0.004	0.00000 n.s.	1.000	7.6712
